# Comparison of three creatinine-based equations to predict adverse outcome in a cardiovascular high-risk cohort: an investigation using the SPRINT research materials

**DOI:** 10.1093/ckj/sfae011

**Published:** 2024-01-19

**Authors:** Insa E Emrich, John W Pickering, Felix Götzinger, Rafael Kramann, Michael Kunz, Lucas Lauder, Vasilios Papademetriou, Michael Böhm, Gunnar H Heine, Felix Mahfoud

**Affiliations:** Saarland University Medical Center, Department of Internal Medicine III, Cardiology, Angiology, and Intensive Care Medicine, Homburg, Germany; Saarland University, Faculty of Medicine, Homburg/Saarbrücken, Germany; Department of Medicine, University of Otago Christchurch and Emergency Care Foundation, Christchurch Hospital, Christchurch, New Zealand; Saarland University Medical Center, Department of Internal Medicine III, Cardiology, Angiology, and Intensive Care Medicine, Homburg, Germany; Saarland University, Faculty of Medicine, Homburg/Saarbrücken, Germany; Division of Nephrology and Clinical Immunology, RWTH Aachen University, Aachen, Germany; Institute of Experimental Medicine and Systems Biology, RWTH Aachen University, Aachen, Germany; Department of Internal Medicine, Nephrology and Transplantation, Erasmus Medical Center, Rotterdam, The Netherlands; Saarland University Medical Center, Department of Internal Medicine III, Cardiology, Angiology, and Intensive Care Medicine, Homburg, Germany; Saarland University, Faculty of Medicine, Homburg/Saarbrücken, Germany; Saarland University Medical Center, Department of Internal Medicine III, Cardiology, Angiology, and Intensive Care Medicine, Homburg, Germany; Saarland University, Faculty of Medicine, Homburg/Saarbrücken, Germany; Department of Veterans Affairs and Georgetown University Medical Centers, Washington DC, USA; Saarland University Medical Center, Department of Internal Medicine III, Cardiology, Angiology, and Intensive Care Medicine, Homburg, Germany; Saarland University, Faculty of Medicine, Homburg/Saarbrücken, Germany; Saarland University, Faculty of Medicine, Homburg/Saarbrücken, Germany; Agaplesion Markus Krankenhaus, Department of Nephrology, Frankfurt am Main, Germany; Saarland University Medical Center, Department of Internal Medicine III, Cardiology, Angiology, and Intensive Care Medicine, Homburg, Germany; Saarland University, Faculty of Medicine, Homburg/Saarbrücken, Germany

**Keywords:** cardiovascular risk prediction, CKD-EPI 2009 equation, CKD-EPI 2021 equation, classification, estimated glomerular filtration rate, European Kidney Function Consortium (EKFC) equation

## Abstract

**Background:**

Novel creatinine-based equations have recently been proposed but their predictive performance for cardiovascular outcomes in participants at high cardiovascular risk in comparison to the established CKD-EPI 2009 equation is unknown.

**Method:**

In 9361 participants from the United States included in the randomized controlled SPRINT trial, we calculated baseline estimated glomerular filtration rate (eGFR) using the CKD-EPI 2009, CKD-EPI 2021, and EKFC equations and compared their predictive value of cardiovascular events. The statistical metric used is the net reclassification improvement (NRI) presented separately for those with and those without events.

**Results:**

During a mean follow-up of 3.1 ± 0.9 years, the primary endpoint occurred in 559 participants (6.0%). When using the CKD-EPI 2009, the CKD-EPI 2021, and the EKFC equations, the prevalence of CKD (eGFR <60 ml/min/1.73 m^2^ or >60 ml/min/1.73 m^2^ with an ACR ≥30 mg/g) was 37% vs. 35.3% (*P *= 0.02) vs. 46.4% (*P *< 0.001), respectively. The corresponding mean eGFR was 72.5 ± 20.1 ml/min/1.73 m^2^ vs. 73.2 ± 19.4 ml/min/1.73 m^2^ (*P *< 0.001) vs. 64.6 ± 17.4 ml/min/1.73 m^2^ (*P *< 0.001). Neither reclassification according to the CKD-EPI 2021 equation [CKD-EPI 2021 vs. CKD-EPI 2009: NRIevents: −9.5% (95% confidence interval (CI) −13.0% to −5.9%); NRInonevents: 4.8% (95% CI 3.9% to 5.7%)], nor reclassification according to the EKFC equation allowed better prediction of cardiovascular events compared to the CKD-EPI 2009 equation (EKFC vs. CKD-EPI 2009: NRIevents: 31.2% (95% CI 27.5% to 35.0%); NRInonevents: −31.1% (95% CI −32.1% to −30.1%)).

**Conclusion:**

Substituting the CKD-EPI 2009 with the CKD-EPI 2021 or the EKFC equation for calculation of eGFR in participants with high cardiovascular risk without diabetes changed the prevalence of CKD but was not associated with improved risk prediction of cardiovascular events for both those with and without the event.

## Graphical Abstract

**Graphical Abstract:**
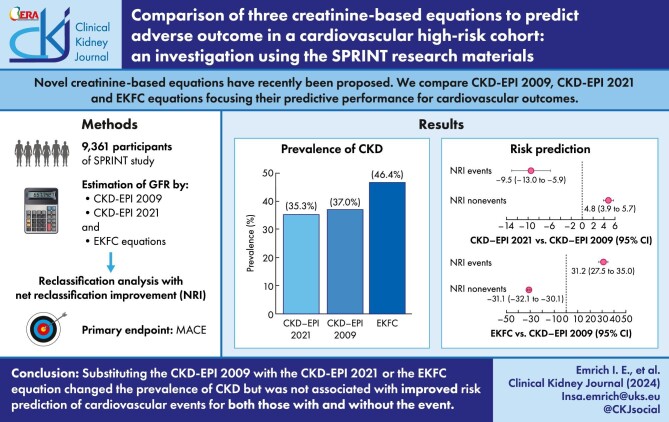

KEY LEARNING POINTS **What was known**:

- Glomerular filtration rate is an established independent predictor of adverse outcome in a broad range of patients.
- Different equations are available to estimate glomerular filtration rate, but whether they led to better risk prediction compared to the established CKD-EPI 2009 equation is not known.
- Implementation of novel equations may cause confusion outside nephrology, which may hamper rather than encourage their use in daily clinical practice.

**This study adds**:

- In this analysis, prevalence of CKD changed according to the used equation.
- Neither the EKFC nor the CKD-EPI 2021 equation led to a better risk prediction in this cohort of high cardiovascular risk patients without diabetes.

**Potential impact**:

- Changing established equations in daily clinical care should be performed with caution: risks and benefits have to be conscientiously considered.

## INTRODUCTION

Glomerular filtration rate (GFR)is used to assess exocrine kidney function and to diagnose and classify chronic kidney disease (CKD). Additionally, GFR is an established independent predictor of all-cause death, cardiovascular diseases, and cardiovascular mortality in a broad range of patients [1]. Current 2012 Kidney Disease: Improving Global Outcomes (KDIGO) guidelines recommended using the CKD-EPI 2009 GFR creatinine equation [2] for estimation of GFR [3]. Only in specific circumstances, GFR estimation with equations based on serum cystatin C level is recommended [3]. The CKD-EPI 2009 creatinine equation integrated not only serum creatinine, but also age, sex, and race [4]. Race, however, has been claimed to represent a social rather than a biological construct. The CKD-EPI Collaboration has therefore recently revised their CKD-EPI 2009 creatinine equation and provided an alternative formula which abstains from using the race criterion (CKD-EPI 2021 creatinine equation) [5]. At the same time, the European Kidney Function Consortium (EKFC) published an alternative equation, which can be used for a broader age range and does not include race [6]. GFR below 60 ml/min/1.73 m^2^ is a trigger for CKD definition. The use of different eGFR equations may affect the prevalence of CKD in the general population. The Systolic Blood Pressure Interventional Trial (SPRINT) has recruited a large number of non-diabetic participants at high risk of a cardiovascular event from the United States, including Puerto Rico [7]. Amongst participants of SPRINT, the present study aimed to compare differences in estimation of CKD prevalence, in KDIGO GFR category allocation and in their association with adverse cardiovascular outcomes when applying the CKD-EPI 2009 creatinine equation, the CKD-EPI 2021 creatinine equation, and the EKFC equation, respectively.

## MATERIALS AND METHODS

We performed a post-hoc analysis of SPRINT [7]. The detailed design and primary results of SPRINT have been published elsewhere [7]. In brief, SPRINT was a randomized, controlled, open-label, multicenter trial of intensive versus standard blood-pressure control conducted in the United States and Puerto Rico from 2010 to 2015 (NCT01206062).

### Population

Participants from the United States, including Puerto Rico, were 50 years or older and at high risk for cardiovascular events with systolic blood pressure (SBP) of 130 to 180 mmHg. They were randomized to a SBP target of <120 mmHg (intensive-treatment group) or <140 mmHg (standard-treatment group). The detailed inclusion and exclusion criteria were previously published [7]. A high risk for cardiovascular events was defined as the presence for a clinical or subclinical cardiovascular disease other than stroke, CKD with an eGFR according to the Modification of Diet in Renal Disease (MDRD) equation of 20 to <60 ml/min/1.73 m^2^ or 10-year risk for cardiovascular disease of ≥15% according to the Framingham risk score. Participants with diabetes mellitus, proteinuria >1 g/day or albumin-to-creatinine ratio (ACR) >600 mg/g, polycystic kidney disease, prior stroke or transient ischemic attack, symptomatic heart failure or left ventricular ejection fraction <35% were excluded. Between November 2010 and March 2013, 9361 participants were enrolled. All participants provided written informed consent. The institutional review board of each participating study site approved the study.

### Exposures

CKD was defined as eGFR≤60 ml/min/1.73 m^2^ or eGFR>60 ml/min/1.73 m^2^ with an ACR ≥30 mg/g. GFR was estimated according to the 2009 CKD-EPI [2], 2021 CKD-EPI [5], and EKFC equations [6] (Table S5, see section 5 of the supplement). Participants were classified according to the 2012 KDIGO guidelines [3], in the following eGFR categories: G1 (eGFR≥90 ml/min/1.73 m^2^), G2 (eGFR 89-60 ml/min/1.73 m^2^), G3a (eGFR 59-45 ml/min/1.73 m^2^), G3b (eGFR 44-30 ml/min/1.73 m^2^), G4 (eGFR 29-15 ml/min/1.73 m^2^) and G5 (eGFR<15 ml/min/1.73 m^2^), respectively.

### Endpoints

The primary endpoint was the composite of myocardial infarction, acute coronary syndrome not resulting in myocardial infarction, stroke, acute decompensated heart failure or death from cardiovascular causes, whichever occurred first. This primary endpoint was directly taken from the original analysis and has not been modified. Subgroup analyses included, beside others, sex and race. On 20 August 2015, the blood-pressure intervention stopped prematurely due to intervention benefit, resulting in a median follow-up of 3.26 years [7].

### Statistical analysis

Categorical variables are presented as absolute numbers and percentages of participants. Continuous data are expressed as means ± standard deviation (or median [interquartile range, IQR] in case of skewed distribution).

Because the CKD-EPI 2009 uses only two categories of race, Black and White, we adopted the SPRINT categorization of all non-Black race as White.

To illustrate the differences between the three equations, density plots and Bland–Altman plots were constructed. Next, we conducted competing risks regression analyses including adjustment for all three equations. The baseline model for competing risk analyses included SBP, diastolic blood pressure (DBP), smoking status, aspirin use, body mass index, high density cholesterol, log transformed triglyceride, total cholesterol, serum glucose, ACR, and study arm. Models are compared with the Akaike information criterion (AIC), the lower the better.

To demonstrate the ability of each of the three equations to classify participants to the KDIGO GFR category that best reflects their risk of reaching the primary endpoint, classification tables are presented. We present two tables that assess the difference between the CKD-EPI 2009 and the CKD-EPI 2021 equation for participants with a cardiovascular event and those with no event and two tables to assess the difference between the CKD-EPI 2009 and the EKFC equation. Subgroup analysis for gender can be found in the supplement. The statistical metric used in each case is the net reclassification improvement (NRI) presented separately for those with and those without events.

NRIevents is the difference between the proportion of participants who reached the primary endpoint who moved to a lower KDIGO GFR (or higher CKD risk) category (lower absolute eGFR) and the proportion of participants reaching the primary endpoint who moved to a higher KDIGO GFR (or lower CKD risk) category (higher absolute eGFR). NRInonevents is the difference between the proportion of participants who did not reach the primary endpoint who moved to a higher KDIGO GFR (or lower CKD risk) category (higher absolute eGFR) and the proportion of participants who did not reach the primary endpoint who moved to a lower KDIGO GFR (or higher CKD risk) category (lower absolute eGFR) [8]. For both the NRIevents and NRInonevents, a positive value represents improved classification (better association with the outcomes). The 95% confidence intervals (CI) for NRIevents and NRInonevents were produced using the bootstrap method. NRI metrics were calculated for the reclassification to different KDIGO GFR categories by eGFR according to the CKD-EPI 2009 equation compared with eGFR according to the CKD-EPI 2021 equation and eGFR according to the EKFC equation. For GFR estimation according to the EKFC equation, the Q value corresponding to the median serum creatinine values for the age- and sex-specific healthy population based on the original development data set has been used [6].

The principal aim of this analysis was showing the predictive performance of the different creatinine-based equations for adverse cardiovascular outcomes.

## RESULTS

SPRINT recruited 9631 participants. A total of 323 individuals had no baseline serum creatinine measurement, which precluded GFR estimation with either CKD-EPI 2009, CKD-EPI 2021, or EKFC equations. Thus, 9308 participants were included into the present analysis.

The mean age was 67.9 ± 9.4 years, 35.5% were female, 29.9% were Black, 10.5% Hispanic, 57.8% White, and 1.9% Others. The mean SBP/DBP was 140 ± 16 mmHg/78 ± 12 mmHg. Further baseline characteristics are summarized in Table 1.

**Table 1:** Baseline characteristics.

Age [years]	67.9 ± 9.4
Gender (female, *n* [%])	3302 (35.5%)
SBP [mmHg]	140 ± 16
DBP [mmHg]	78 ± 12
BMI [kg/m^2^]	30 ± 6
Total cholesterol [mg/dl]	190 ± 41
Serum glucose [mg/dl]	99 ± 14
High density cholesterol [mg/dl]	53 ± 14
Serum triglyceride [mg/dl]	126 ± 91
ACR [mg/g]	43 [1.36–5000.0]
Serum creatinine [mg/dl]	1.07 ± 0.34
Risk10Yrs [%]	20.1 ± 10.9
Statin use (*n* [%])	4036 (43.6%)
Aspirin use (*n* [%])	4737 (51.0%)
Antihypertensive treatment (*n* [%])	8428 (90.5%)
Smoking (*n* [%])	1237 (13.3%)
Prevalent cardiovascular disease (*n* [%])	1553 (16.7%)
Black (*n* [%])	2783 (29.9%)
Hispanic (*n* [%])	975 (10.5%)
White (*n* [%])	5376 (57.8%)
Other (*n* [%])	174 (1.9%)
eGFR MDRD [ml/min/1.73 m^2^]	71.8 ± 20.6
eGFR CKD-EPI 2009 [ml/min/1.73 m^2^]	72.5 ± 20.1
eGFR CKD-EPI 2021 [ml/min/1.73 m^2^]	73.2 ± 19.4
eGFR EKFC [ml/min/1.73 m^2^]	64.6 ± 17.4

ACR: albumin-to-creatinine ratio; BMI: body mass index; DBP: diastolic blood pressure; definition of smoking: current smoking or in the past 30 days; eGFR: estimated glomerular filtration rate; Risk10Yrs: Framingham estimation of 10-year cardiovascular disease risk; SBP: systolic blood pressure.

The mean estimates of GFR differed according to the eGFR equation used with 72.5 ± 20.1 ml/min/1.73 m^2^ for CKD-EPI 2009 versus 73.2 ± 19.4 ml/min/1.73 m^2^ for CKD-EPI 2021 (*P *< 0.001) versus 64.6 ± 17.4 ml/min/1.73 m^2^ for EKFC (*P *< 0.001), respectively. Of note, in the original publication, renal function was estimated with the MDRD equation resulting in a mean eGFR of 71.8 ± 20.6 ml/min/1.73 m^2^.

**Figure 1:**
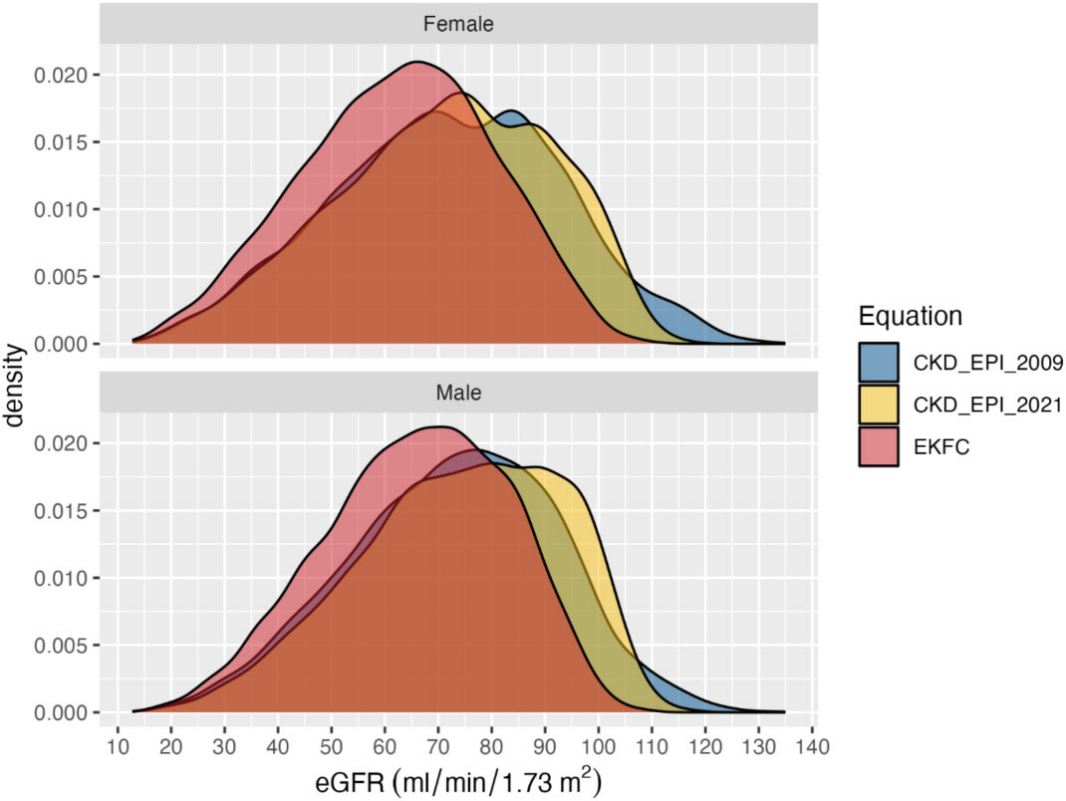
Comparison of the three equations separated by gender: GFR estimated by the EKFC equation was less skewed and shifted to lower eGFR than GFR estimated by the CKD-EPI 2009 equation (mean difference −7.9 [95%CI: −7.8 to −8.0] ml/min/1.73 m^2^). The difference between eGFR according to the CKD-EPI 2009 equation and the CKD-EPI 2021 equation was less pronounced (mean difference 0.7 [95%CI: 0.6 to 0.8] ml/min/1.73 m^2^).

When comparing the three equations used herein, GFR estimated by the EKFC equation was less skewed and shifted to lower eGFR than GFR estimated by the CKD-EPI 2009 equation (mean difference −7.9 [95%CI: −7.8 to −8.0]ml/min/1.73 m^2^), Fig. 1 (male vs. female participants). The difference between eGFR according to the CKD-EPI 2009 equation and the CKD-EPI 2021 equation was less pronounced (mean difference 0.7 [95%CI: 0.6 to 0.8]ml/min/1.73 m^2^) (Fig. 1, male vs. female participants).

**Figure 2:**
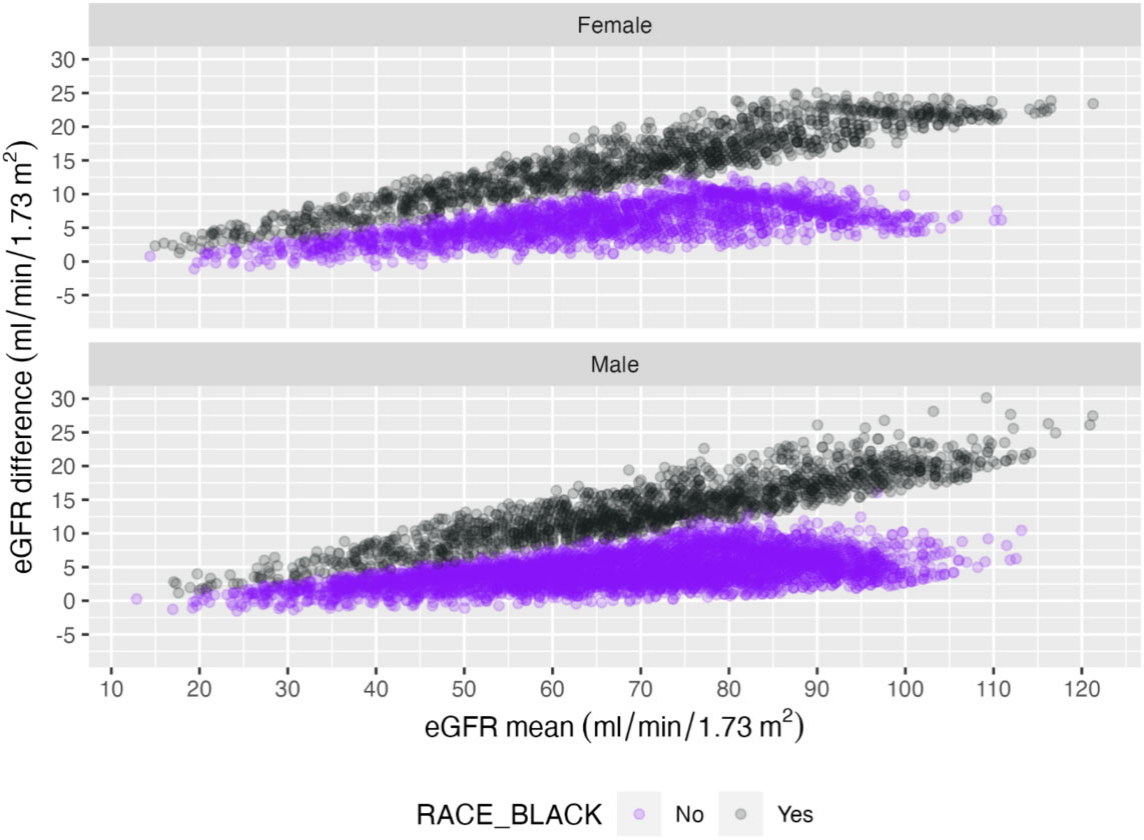
Bland–Altman plots: The y-axis is the eGFR difference: CKD-EPI 2009–EKFC (positive eGFR difference: GFR estimation with CKD-EPI 2009 is overall higher). The x-axis is the mean of the two eGFRs. Nearly all individual eGFR calculated by the CKD-EPI 2009 equation were higher than respective eGFR calculated by the EKFC equation. This difference increased with higher eGFR; Black participants had a lower difference than non-Black participants.

**Figure 3:**
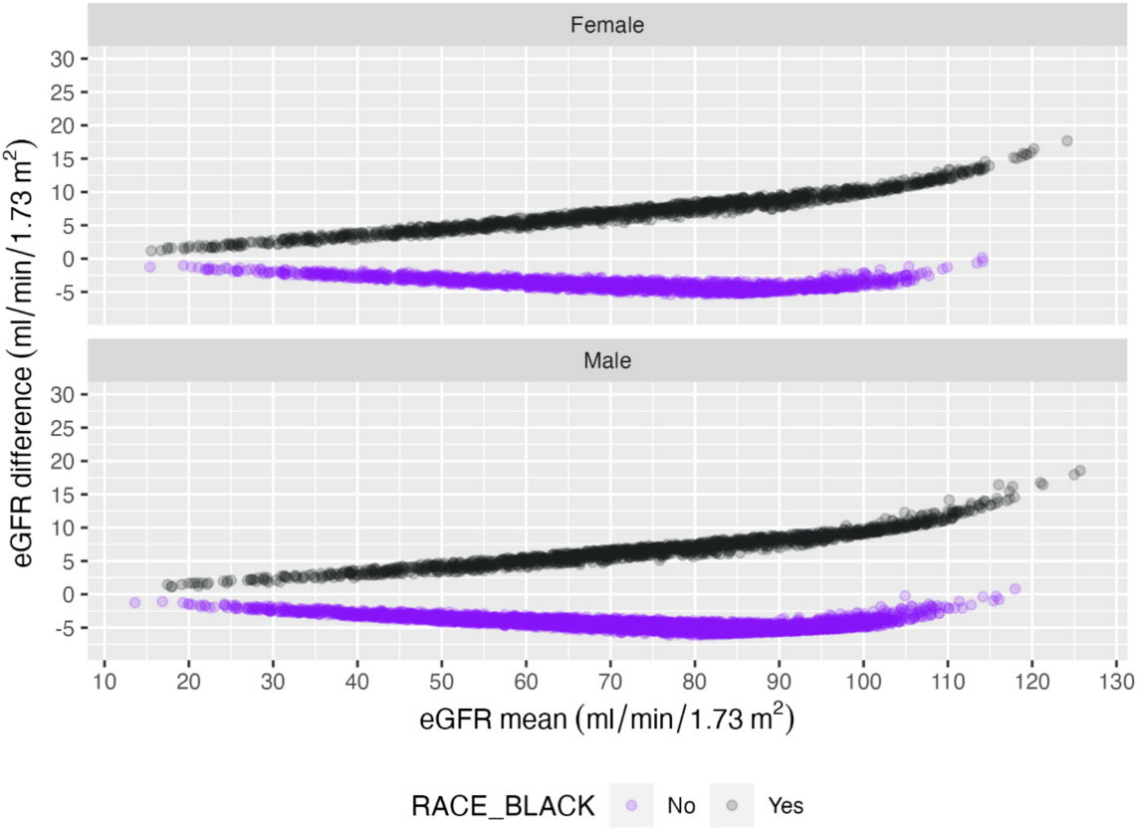
Bland–Altman plots: The y-axis is the eGFR difference: CKD-EPI 2009–CKD-EPI 2021 (positive eGFR difference: GFR estimation with CKD-EPI 2009 is overall higher). The x-axis is the mean of the two eGFRs. Compared with eGFR calculated by the CKD-EPI 2009 equation, the eGFR calculated by the CKD-EPI 2021 equation was higher for almost all non-Black, but lower for all Black participants.

Bland–Altman plots compared the mean of two estimating equations with their difference for each sex and race. Nearly all individual eGFR calculated by the CKD-EPI 2009 equation were higher than respective eGFR calculated by the EKFC equation. This difference increased with higher eGFR; Black participants had a lower difference than non-Black participants (Fig. 2). Compared with eGFR calculated by the CKD-EPI 2009 equation, the eGFR calculated by the CKD-EPI 2021 equation was higher for almost all non-Black, but lower for all Black participants (Fig. 3).

Table 2 provides an overview of the KDIGO GFR categories according to the three different equations. The CKD-EPI 2021 equation compared with the CKD-EPI 2009 equation reclassified 7.0% of the participants to a more advanced KDIGO GFR category and 12.2% to a less advanced category. The EKFC equation compared with the CKD-EPI 2009 equation reclassified 31.1% to a more advanced KDIGO GFR category and <0.1% (*n* = 1) to a less advanced category.

**Table 2:** KDIGO GFR categories according to the three different equations.

	CKD-EPI 2009	CKD-EPI 2021	EKFC
G1; *n* [%]	1870 (20.1%)	2082 (22.4%)	596 (6.4%)
G2; *n* [%]	4914 (52.8%)	4894 (52.6%)	5119 (55.0%)
G3a; *n* [%]	1611 (17.3%)	1482 (15.9%)	2218 (23.8%)
G3b; *n* [%]	743 (8.0%)	690 (7.4%)	1118 (12.0%)
G4; *n* [%]	168 (1.8%)	158 (1.7%)	253 (2.7%)
G5; *n* [%]	2 (0%)	2 (0%)	4 (0%)
<60 ml/min/1.73 m^2^; *n* [%]	2524 (27.1%)	2332 (25.1%)	3593 (38.6%)

GFR: glomerular filtration rate. Note eGFR <20 ml/min/1.73 m**^2^** by the MDRD equation was an exclusion criterion for SPRINT, hence very few patients in KDIGO GFR category G5 by these other equations.

Measurement of ACR was available for 8886 (95.4%) of the participants. ACR of ≥30 mg/g was found in 1730 participants. When considering both eGFR and ACR for the definition of CKD (eGFR <60 ml/min/1.73 m^2^ independently of ACR or eGFR ≥60 ml/min/1.73 m^2^ plus ACR ≥30 mg/g), 37.0% were diagnosed with CKD by the CKD-EPI 2009, 35.3% (Difference −1.7% [95%CI: −0.3% to −3.1%], *P *= 0.02) by the CKD-EPI 2021, and 46.4% (Difference 9.4% [95%CI: 7.9% to 10.8%], *P *< 0.001) by EKFC, respectively.

During a mean follow-up of 3.1 ± 0.9 years, the primary endpoint was reached by 559 participants (6.0%). In a competing risk model, additional adjusting for eGFR according to the EKFC equation (AIC 9483.67) to the baseline model (AIC 9501.07) added most information to the outcome prediction, followed by the CKD-EPI 2009 equation (AIC 9484.92) and the CKD-EPI 2021 equation (AIC 9488.66). However, the diagnostic value of the addition of any eGFR equation was minimal with no changes in the AUCs and small changes in Brier skill indicating very slightly improved calibration (see section 3 of the supplement). This was similar for subgroup analysis considering race and sex.

The number of participants reaching the primary endpoint across all KDIGO GFR categories are depicted in Table 3. Survival curves for the primary outcome according to all three equations are presented in the supplement (see section 2 of the supplement). Among 559 participants reaching the primary endpoint, 26 were reclassified from CKD-EPI 2009 KDIGO GFR category to a more advanced category by the CKD-EPI 2021 equation and 79 to a less advanced category, yielding a NRIevents of −9.5% (95%CI −13.0% to −5.9%). This represents poorer performance. Of the 8749 participants without an event, 630 participants moved to a more advanced KDIGO GFR category according to the CKD-EPI 2021 equation and 1054 to a less advanced KDIGO GFR category, resulting in a NRInonevents of 4.8% (95%CI 3.9% to 5.7%); this represents improved performance (Table 3A).

**Table 3A:** Reclassification of KDIGO GFR categories when utilizing CKD-EPI 2021 instead of CKD-EPI 2009.

	Events							No events					
	NRIevents: −9.5% (95% CI: −13% to −5.9%)							NRInonevents: 4.8% (95% CI: 3.9% to 5.7%)					
	CKD-EPI 2021							CKD-EPI 2021					
	G1	G2	G3a	G3b	G4	G5		G1	G2	G3a	G3b	G4	G5
CKD-EPI 2009							CKD-EPI 2009						
G1	68	**11**	0	0	0	0	G1	1466	*325*	0	0	0	0
G2	*31*	210	**7**	0	0	0	G2	**517**	3950	*199*	0	0	0
G3a	0	*23*	90	**4**	0	0	G3a	0	**375**	1032	*87*	0	0
G3b	0	0	*18*	68	**4**	0	G3b	0	0	**136**	499	*18*	0
G4	0	0	0	*7*	18	0	G4	0	0	0	**25**	117	*1*
G5	0	0	0	0	0	0	G5	0	0	0	0	**1**	1

Participants are classified according to their KDIGO GFR category according to the GFR estimation following the CKD-EPI 2009 and the CKD-EPI 2021 equation, separated for ‘events’ and ‘no events’. All those written in bold are reclassified to a more suitable category according to their ‘event—no event status’, all those written in italics are reclassified falsely according to their ‘event—no event status’. G1: eGFR ≥90 ml/min/1.73 m^2^; G2: eGFR 60–89 ml/min/1.73 m^2^; G3a: eGFR 45–59 ml/min/1.73 m^2^; G3b: 30–44 ml/min/1.73 m^2^; G4: eGFR 15–29 ml/min/1.73 m^2^; G5: eGFR <15 ml/min/1.73 m^2^. CI: confidence interval; eGFR: estimated glomerular filtration rate; NRI: net reclassification improvement.

In contrast, among 559 participants reaching the primary endpoint, 175 were reclassified from the CKD-EPI 2009 KDIGO GFR category to a more advanced KDIGO GFR category by the EKFC equation and none to a less advanced KDIGO GFR category, yielding a NRIevents of 31.2% (95%CI 27.5% to 35.0%); this represents improved performance. Of the 8749 participants without an event, 2720 participants were reclassified to a more advanced KDIGO GFR category by the EKFC equation and only one to a less advanced KDIGO GFR category, resulting in a NRInonevents of −31.1% (95%CI −32.1% to −30.1%); this represents poorer performance, Table 3B.

**Table 3B:** Reclassification of KDIGO GFR categories when utilizing EKFC instead of CKD-EPI 2009.

	Events						No events					
	NRIevents: 31.2% (95% CI: 27.5% to 35.0%)						NRInonevents: −31.1% (95% CI: −32.1% to −30.1%)					
	EKFC						EKFC					
	G1	G2	G3a	G3b	G4	G5	G1	G2	G3a	G3b	G4	G5
CKD-EPI 2009												
G1	18	**61**	0	0	0	0	578	*1213*	0	0	0	0
G2	0	189	**59**	0	0	0	0	3656	*1010*	0	0	0
G3a	0	0	78	**39**	0	0	0	0	1071	*423*	0	0
G3b	0	0	0	74	**16**	0	0	0	0	581	*72*	0
G4	0	0	0	0	25	0	0	0	0	**1**	140	*2*
G5	0	0	0	0	0	0	0	0	0	0	0	2

Participants are classified according to their KDIGO GFR category according to the GFR estimation following the CKD-EPI 2009 and the EKFC equation, separated for ‘events’ and ‘no events’. All those written in bold are reclassified to a more suitable category according to their ‘event—no event status’, all those written in italic are reclassified falsely according to their ‘event—no event status’. G1: eGFR ≥90 ml/min/1.73 m^2^; G2: eGFR 60–89 ml/min/1.73 m^2^; G3a: eGFR 45–59 ml/min/1.73 m^2^; G3b: 30–44 ml/min/1.73 m^2^; G4: eGFR 15–29 ml/min/1.73 m^2^; G5: eGFR <15 ml/min/1.73 m^2^. CI: confidence interval; eGFR: estimated glomerular filtration rate; NRI: net reclassification improvement.

When integrating ACR within the definition of CKD, among the 547 participants reaching the primary endpoint, 13 were reclassified to have CKD according to the CKD-EPI 2009 equation to not having CKD according to the CKD-EPI 2021 equation. Three were reclassified from not having CKD to having CKD according to the CKD-EPI 2021 equation, resulting in a NRIevents of 1.8% (95%CI 0.5% to 3.4%); this represents improved performance. Of the 8339 participants without an event, 295 moved from having CKD to not having CKD and 158 from not having CKD to having CKD, yielding a NRInonevents of −1.6% (95%CI −2.1% to −1.2%); this represents poorer performance (Table 3C). Compared to those without CKD before and after reclassification, the odds of those who were reclassified from not having CKD to CKD did not increase [OR 0.4 (95%CI 0.1 to 1.3), see section 4 of the supplement].

**Table 3C:** Reclassification of CKD status when utilizing CKD-EPI 2021 instead of CKD-EPI 2009.

	Events		No events	
	NRIevents: 1.8% (95% CI: 0.5% to 3.4%)		NRInonevents: −1.6% (95% CI: −2.1% to −1.2%)	
	CKD-EPI 2021		CKD-EPI 2021	
	CKD	NON-CKD	CKD	NON-CKD
CKD-EPI 2009				
CKD	295	*13*	2759	**295**
NON-CKD	**3**	236	*158*	5127

Participants are classified according to the definition of CKD and NON-CKD according to the GFR estimation following the CKD-EPI 2009 and the CKD-EPI 2021 equation, separated for ‘events’ and ‘no events’. All those written in bold are reclassified to a more suitable group according to their ‘event—no event status’, all those written in italic are reclassified falsely according to their ‘event—no event status’. CI: confidence interval; CKD: chronic kidney disease, defined as eGFR <60 ml/min/1.73 m^2^ or eGFR >60 ml/min/1.73 m^2^ and albumin-to-creatinine ratio ≥30 mg/g; NRI: net reclassification improvement.

Among the 547 participants reaching the primary endpoint, no one was reclassified from having CKD according to the CKD-EPI 2009 equation to not having CKD according to the EKFC equation and 42 were reclassified from not having CKD to having CKD according to the EKFC equation, resulting in a NRIevents of −7.7% (95%CI −10.1% to −5.7%); this represents poorer performance. Of the 8339 participants without an event, no one moved from CKD to non-CKD and 785 participants moved from non-CKD to CKD according to the EKFC equation, resulting in a NRInonevents of 9.4% (95%CI 8.8% to 10.1%); this represents improved performance (Table 3d). Compared to those without CKD before and after reclassification, the odds of those who were reclassified from not having CKD to CKD had a small increase [OR 1.2 (95%CI 0.9 to 1.7), see section 4 of the supplement].

**Table 3D:** Reclassification of CKD status when utilizing EKFC instead of CKD-EPI 2009.

	Events		No events	
	NRIevents: −7.7% (95% CI: −10.1% to −5.7%)		NRInonevents: 9.4% (95% CI: 8.8% to 10.1%)	
	EKFC		EKFC	
	CKD	NON-CKD	CKD	NON-CKD
CKD-EPI 2009				
CKD	308	0	3054	0
NON-CKD	**42**	197	*785*	4500

Participants are classified according to the definition of CKD and NON-CKD according to the GFR estimation following the CKD-EPI 2009 and the EKFC equation, separated for ‘events’ and ‘no events’. All those written in bold are reclassified to a more suitable group according to their ‘event—no event status’, all those written in italics are reclassified falsely according to their ‘event—no event status’. CI: confidence interval; CKD: chronic kidney disease, defined as eGFR <60 ml/min/1.73 m^2^ or eGFR >60 ml/min/1.73 m^2^ and albumin-to-creatinine ratio ≥30 mg/g; EKFC: European Kidney Function Consortium; NRI: net reclassification improvement.

Subgroup analyses for sex are presented in the supplement (Supplementary Tables S1 and S2, see section 1 of the supplement).

## DISCUSSION

The main findings from this post-hoc analysis of the SPRINT trial are:

- calculating eGFR using the EKFC instead of the CKD-EPI 2009 equation resulted in a lower mean eGFR and a reclassification of a net 31.1% to a more advanced KDIGO GFR category, resulting in a net 9.4% higher prevalence of CKD (46.4% versus 37.0%);
- the differences of the CKD-EPI 2021 and the CKD-EPI 2009 equation were less prominent, but with a trend of reclassification towards a less advanced KDIGO GFR category compared to EKFC; and
- despite small improvements in the regression model, the reclassification approach did not improve the risk prediction of the eGFR categories for cardiovascular events for both those with and without the event. When using EKFC instead of CKD-EPI 2009, those with the event were classified into a lower GFR (or higher CKD risk) category (positive NRIevents). Those without the event were also classified into a lower GFR (or higher CKD risk) category (negative NRInonevents).

Multiple studies identified CKD as an independent cardiovascular risk factor and indicated an association between decreased GFR and increased risk of cardiovascular events [9]. Therefore, adequate estimation of GFR, to enable precise risk classification according to the KDIGO GFR categories remains an important objective of any new GFR equation before its clinical implementation can be recommended [3]. The recent KDIGO clinical practice guidelines for the evaluation and management of CKD recommend the use of the CKD-EPI 2009 equation [2], which has been shown to be more accurate, precise, and less biased than previously established equations as MDRD [10] or Cockroft–Gault [11] equations. The guidelines also state the importance of appraising best risk prediction [3]. Both the novel CKD-EPI 2021 [5] and the EKFC equations [6] were published after the KDIGO clinical practice guidelines for the evaluation and management of CKD.

Because race is a social and political rather than a biological construct, the CKD-EPI 2021 [5] equation was introduced in the United States to remove race as a variable for estimating GFR. In comparison to CKD-EPI 2009 [2], coefficients for intercept, age, sex, and creatinine concentration have been revised. A recently published study showed that if the race variable is omitted from conventional creatinine-based equations without mathematical adaption of the other coefficients, there is a greater systematic underestimation of GFR among Black adults [12]. This is related to the fact that individuals with African ancestry have on average higher serum creatinine concentrations than non-Black [12]. Serum cystatin C-based GFR estimations, on the other hand, were shown to be independent of race or ancestry [12]. Because measurement of serum cystatin C for estimating GFR is relatively expensive and not widely available, the use of the new creatinine-based CKD-EPI 2021 equation [5] with the changed coefficients has recently been recommend by the National Kidney Foundation [13] and American Society of Nephrology Task Force for US adults [14]. Employing the CKD-EPI 2021 equation in 246.6 million people in the United States was associated with a lower estimate of the CKD prevalence when compared with the CKD-EPI 2009 equation (26.9 million vs. 29.6 million) [5]. A similar decrease in estimated CKD prevalence was found by an analysis of 1.6 million Swedish adults with serum creatinine measurement from routine healthcare [15]. A total of 36.2% of the participants were reclassified to a less advanced KDIGO GFR category by the CKD-EPI 2021 equation. These reclassified participants had a higher risk of all-cause mortality, cardiovascular mortality, and major adverse cardiovascular events, but a lower risk of kidney failure compared with participants who were not reclassified [15]. Based on these findings, it has been proposed by a European consensus group not to adopt the CKD-EPI 2021 equation to estimate GFR in Europe [16]. Instead, it was suggested to use (i) serum creatinine in line with weight and height as additional covariates to account for interindividual differences in muscle mass; (ii) serum cystatin C (alone or in combination with creatinine) for estimation of GFR; or (iii) the EKFC equation instead [16]. The EKFC equation [6] is an advancement of the FAS (full age spectrum) equation [17], developed to improve GFR estimation during transition from adolescence to adulthood to ameliorate overestimation of GFR in young adults. However, Black individuals were not included in the development data set, which mainly comprised individuals from Europe. As a common feature, both equations—the EKFC and the FAS—are creatinine-based and use Q-values, which are sex- and age-specific median creatinine concentrations from healthy subjects to assess GFR. For our analysis, we used the original EKFC equation published in November 2020 [6]. Meanwhile, an adapted EKFC equation was proposed that uses standard sex- and age-adjusted Q-values for Caucasian European females and males and adjusted these Q-values for other populations, namely, Black female Africans, Black male Africans, Black female Europeans, and Black male Europeans [18]. This factor was not available when designing our analyses. In a cohort of 18 893 participants with measurement of GFR, the EKFC equation adjusted for the population-dedicated Q-values had the best performance compared with the CKD-EPI 2009 equation without race correction and the CKD-EPI 2021 equation. Reclassification analyses, however, have yet to be published [18]. Recently, a specific adapted Q-value for the US Black population has been published, but has not been validated in cohort studies yet [19]. Hitherto, an adapted Q-value for Hispanics has not been determined. Additionally, in three Chinese cohort studies the EKFC equation has been compared to the CKD-EPI 2009 equation without the use of adapted Asian-specific Q values [20–22]. A study in 612 elderly participants illustrated that the EKFC equation was not significantly better than the CKD-EPI 2009 equation [21]. In 160 Chinese CKD patients there was not a clinically meaningful difference in the performance of the Asian-modified CKD-EPI equation and the EKFC equations [22]. In a further analysis of 3692 Chinese participants a better performance of the EKFC equation compared to the 2009 CKD-EPI equation has been described [20]. This study group published in the supplement Chinese-specific Q values that were overall lower than the used European Q values [20], showing the importance of individual ethnicities-adapted Q values that should be generated in large population databases in the future [23].

In our analysis, the CKD-EPI 2021 equation reclassified the SPRINT's participants to a less advanced KDIGO GFR category; however, this was not associated with improved risk prediction. Participants with and without cardiovascular events were reclassified to a less advanced KDIGO category and/or redefined as not having CKD compared with the CKD-EPI 2009 equation. During the review process of our manuscript, a further analysis of the SPRINT's data set has been published along with results from EMPA-REG OUTCOME and IDNT showing that mean baseline eGFR according to the CKD-EPI 2021 equation was higher in non-Black individuals [mean (SD) difference between the two eGFRcr: 3.7 (1.0)] without significant quantitative impact on the estimated treatment effects on GFR slope or on clinical kidney outcomes [24].

In comparison, reclassification by EKFC was mostly observed into more advanced KDIGO GFR categories and increased the prevalence of CKD by 9.4%. These results were consistent across various subgroups including male vs female participants. Calculation of eGFR using the novel equations EKFC and CKD-EPI 2021 compared with CKD-EPI 2009 was not associated with improved risk prediction for major cardiovascular events in participants at high cardiovascular risk included in the SPRINT trial.

Clinicians should consider the equations’ discrepancy when selecting a respective eGFR equation, as risk prediction may facilitate treatment decisions, dosing of drugs and eventually initiation of renal replacement therapy. Against this background, it should be noted that the publication of different GFR formula in the past years may have caused some confusion. A single equation might facilitate cardiovascular and renal risk prediction. The use of a serum cystatin C-based equation may be one solution as previous studies have indicated good predictive power [25, 26]. Recently a new cystatin C equation has been published without the inclusion of race and sex [27]. Unfortunately, cystatin C measurements are relatively expensive. Furthermore, cystatin C concentrations can be influenced by obesity [28], inflammation [29], steroid intake [30], or thyroid function [31].

The following limitations of the current analysis should be considered: first, only one-third of the participants had CKD at baseline (according to the MDRD equation, used in the original publication) [7]. Hence, participants were classified to KDIGO GFR categories without considering other parameters of kidney damage such as urine sediment abnormalities or markers of tubular disorders. Second, we only considered a single GFR estimation and a single measurement of ACR for the definition of CKD and did not confirm the results with a second analysis. Third, we cannot provide accuracy analyses, as GFR was not measured in SPRINT. Fourth, participants with eGFR <20 ml/min/1.73 m^2^, with proteinuria ≥1 g/g creatinine or albuminuria ≥600 mg/g creatinine, with diabetes, younger than 50 years of age or with prior stroke were excluded. In addition, the SPRINT trial only included participants from the United States, including Puerto Rico, limiting the overall representativeness of the analysed cohort. Fifth, we only considered creatinine-based equations and did not include cystatin C-based ones. Furthermore, as stated above, for the EKFC calculation the Q value from the original development data set has been taken, which has not been adapted for the Black US population or Hispanics, although the development data set as well as the internal and external validation data set included cohorts from the United States (CRIC and ECAC/GENOA study) [6]. As SPRINT was stopped prematurely because of results benefitting the blood pressure intervention group, overall frequency of CV events is limited (6.0% in 3.1 years).

## CONCLUSIONS

Substituting the CKD-EPI 2009 with the CKD-EPI 2021 or the EKFC equation for calculation of eGFR in participants with high cardiovascular risk without diabetes changed the prevalence of CKD but was not associated with improved risk prediction of cardiovascular events for both those with and without the event. Further clinical studies must verify the benefit of these equations. Changing established equations in daily clinical care should be performed with caution: risks and benefits have to be conscientiously considered.

## Supplementary Material

sfae011_Supplemental_File

## Data Availability

Research Materials were received from the NHLBI Biologic Specimen and Data Repository Information Coordinating Center.

## References

[1] Matsushita K , van der Velde M, Astor BC et al. Association of estimated glomerular filtration rate and albuminuria with all-cause and cardiovascular mortality in general population cohorts: a collaborative meta-analysis. Lancet 2010;375:2073–81. 10.1016/S0140-6736(10)60674-5 20483451 PMC3993088

[2] Levey AS , Stevens LA, Schmid CH et al. A new equation to estimate glomerular filtration rate. Ann Intern Med 2009;150:604–12. 10.7326/0003-4819-150-9-200905050-00006 19414839 PMC2763564

[3] Stevens PE , Levin A. Evaluation and management of chronic kidney disease: synopsis of the kidney disease: improving global outcomes 2012 clinical practice guideline. Ann Intern Med 2013;158:825–30. 10.7326/0003-4819-158-11-201306040-00007 23732715

[4] Inker LA , Schmid CH, Tighiouart H et al. Estimating glomerular filtration rate from serum creatinine and cystatin C. N Engl J Med 2012;367:20–29. 10.1056/NEJMoa1114248 22762315 PMC4398023

[5] Inker LA , Eneanya ND, Coresh J et al. New creatinine- and cystatin C–Based equations to estimate GFR without race. N Engl J Med 2021;385:1737–49. 10.1056/NEJMoa2102953 34554658 PMC8822996

[6] Pottel H , Björk J, Courbebaisse M et al. Development and validation of a modified full age spectrum creatinine-based equation to estimate glomerular filtration rate. Ann Intern Med 2021;174:183–91. 10.7326/M20-4366 34280339

[7] Wright JT , Williamson JD, Whelton PK et al. SPRINT Trial. N Engl J Med 2015;373:2103–16. 10.1056/NEJMoa1511939 26551272 PMC4689591

[8] Pickering JW , Endre ZH. New metrics for assessing diagnostic potential of candidate biomarkers. Clin J Am Soc Nephrol 2012;7:1355–64. 10.2215/CJN.09590911 22679181

[9] Go AS , Chertow GM, Fan D et al. Chronic kidney disease and the risks of death, cardiovascular events, and hospitalization. N Engl J Med 2004;351:1296–305. 10.1056/NEJMoa041031 15385656

[10] Levey AS , Bosch JP, Lewis JB et al. A more accurate method to estimate glomerular filtration rate from serum creatinine: a new prediction equation. Ann Intern Med 1999;130:461–70. 10.7326/0003-4819-130-6-199903160-00002 10075613

[11] Cockcroft DW , Gault MH. Prediction of creatinine clearance from serum creatinine. Nephron 1976;16:31–41. 10.1159/000180580 1244564

[12] Hsu C , Yang W, Parikh RV et al. Race, genetic ancestry, and estimating kidney function in CKD. N Engl J Med 2021;385:1750–60. 10.1056/NEJMoa2103753 34554660 PMC8994696

[13] Delgado C , Baweja M, Crews DC et al. A unifying approach for GFR estimation: recommendations of the NKF-ASN Task Force on reassessing the inclusion of race in diagnosing kidney disease. Am J Kidney Dis 2022;79:268–288.e1. 10.1053/j.ajkd.2021.08.003 34563581

[14] Delgado C , Baweja M, Burrows NR et al. Reassessing the inclusion of race in diagnosing kidney diseases: an interim report from the NKF-ASN Task Force. Am J Kidney Dis 2021;78:103–15. 10.1053/j.ajkd.2021.03.008 33845065 PMC8238889

[15] Fu EL , Coresh J, Grams ME et al. Removing race from the CKD-EPI equation and its impact on prognosis in a predominantly White European population. Nephrol Dial Transplant 2023;38:119–28. 10.1093/ndt/gfac197 35689668 PMC9869854

[16] Gansevoort RT , Anders HJ, Cozzolino M et al. What should European nephrology do with the new CKD-EPI equation? Nephrol Dial Transplant 2023;38:1–6. 10.1093/ndt/gfac254 36069913 PMC9869851

[17] Pottel H , Hoste L, Dubourg L et al. An estimated glomerular filtration rate equation for the full age spectrum. Nephrol Dial Transplant 2016;31:798–806. 10.1093/ndt/gfv454 26932693 PMC4848755

[18] Delanaye P , Vidal-Petiot E, Björk J et al. Performance of creatinine-based equations to estimate glomerular filtration rate in white and black populations in Europe, Brazil and Africa. Nephrol Dial Transplant 2023;38:106–18. 10.1093/ndt/gfac241 36002032

[19] Delanaye P , Cavalier E, Pottel H et al. New and old GFR equations: a European perspective. Clin Kidney J 2023;16:1375–83. 10.1093/ckj/sfad039 37664574 PMC10469124

[20] Ma Y , Wei L, Yong Z et al. Validation of the European Kidney Function Consortium equation in Chinese adult population: an equation standing on the shoulders of predecessors. Nephron 2023;1–11. 10.1159/000531030 37315553

[21] Xia F , Hao W, Liang J et al. Applicability of creatinine-based equations for estimating glomerular filtration rate in elderly Chinese patients. BMC Geriatr 2021;21:481. 10.1186/s12877-021-02428-y 34481470 PMC8418712

[22] Zhao L , Li HL, Liu HJ et al. Validation of the EKFC equation for glomerular filtration rate estimation and comparison with the Asian-modified CKD-EPI equation in Chinese chronic kidney disease patients in an external study. Ren Fail 2023;45:2150217. 10.1080/0886022X.2022.2150217 36632770 PMC9848359

[23] Delanaye P , Pottel H. Estimating glomerular filtration rate in China: is the European Kidney Function Consortium (EKFC) equation the solution? Nephron 2023;7:1–4. 10.1159/000531314 37423213

[24] Chaudhari J , Miao S, Lewis JB et al. Impact of using the race-free 2021 CKD-EPI creatinine equation on treatment effects on GFR-based endpoints in clinical trials. Am J Kidney Dis 2024;83:269–72. 10.1053/j.ajkd.2023.05.012 37657637

[25] Emrich IE , Pickering JW, Schöttker B et al. Comparison of the performance of 2 GFR estimating equations using creatinine and cystatin C to predict adverse outcomes in elderly individuals. Am J Kidney Dis 2015;65 636–8. 10.1053/j.ajkd.2014.12.006 25620662

[26] Rogacev KS , Pickering JW, Seiler S et al. The Chronic Kidney Disease Epidemiology Collaboration (CKD-EPI) equation incorporating both cystatin C and creatinine best predicts individual risk: a cohort study in 444 patients with chronic kidney disease. Nephrol Dial Transplant 2014;29:348–55. 10.1093/ndt/gft422 24166454

[27] Pottel H , Björk J, Rule AD et al. Cystatin C–based equation to estimate GFR without the inclusion of race and sex. N Engl J Med 2023;388:333–43. 10.1056/NEJMoa2203769 36720134

[28] Naour N , Fellahi S, Renucci JF et al. Potential contribution of adipose tissue to elevated serum cystatin C in human obesity. Obesity 2009;17:2121–6. 10.1038/oby.2009.96 19360013

[29] Xu Y , Schnorrer P, Proietto A et al. IL-10 controls cystatin C synthesis and blood concentration in response to inflammation through regulation of IFN regulatory factor 8 expression. J Immunol 2011;186:3666–73. 10.4049/jimmunol.1001934 21300820

[30] Risch L , Herklotz R, Blumberg A, Huber AR. Effects of glucocorticoid immunosuppression on serum cystatin C concentrations in renal transplant patients. Clin Chem 2001;47:2055–9. https://pubmed.ncbi.nlm.nih.gov/11673383/11673383

[31] Wiesli P , Schwegler B, Spinas GA et al. Serum cystatin C is sensitive to small changes in thyroid function. Clin Chim Acta 2003;338:87–90. 10.1016/j.cccn.2003.07.022 14637271

