# Supplementary material for: Comparison of three creatinine-based equations to predict adverse outcome in a cardiovascular high-risk cohort: an investigation using the SPRINT research materials
Source: Clin Kidney J. 2024 Jan 19;17(2):sfae011. doi: 10.1093/ckj/sfae011 (PMC10836528; doi:10.1093/ckj/sfae011)
Supplement: sfae011_Supplemental_File [file sfae011_supplemental_file.docx]

**Supplement to: Comparison of three creatinine-based equations to predict adverse outcome in a cardiovascular high-risk cohort:** An investigation using the SPRINT research materials.

Emrich et al.

**Table of Contents**

[1. Confusion matrices 2](#_Toc148017067)

[Table S1A: Reclassification of female participants from CKD-EPI 2009 to CKD-EPI 2021 KDIGO GFR categories 2](#_Toc148017068)

[Table S1B: Reclassification of female participants from CKD-EPI 2009 to EKFC KDIGO GFR categories 3](#_Toc148017069)

[Table S1C: Reclassification of female participants from CKD-EPI 2009 to CKD-EPI 2021 CKD – NON-CKD 3](#_Toc148017070)

[Table S1D: Reclassification of female participants from CKD-EPI 2009 to EKFC CKD – NON-CKD 4](#_Toc148017071)

[Table S2A: Reclassification of male participants from CKD-EPI 2009 to CKD-EPI 2021 KDIGO GFR categories 4](#_Toc148017072)

[Table S2B: Reclassification of male participants from CKD-EPI 2009 to EKFC KDIGO GFR categories 5](#_Toc148017073)

[Table S2C: Reclassification of male participants from CKD-EPI 2009 to CKD-EPI 2021 CKD – NON-CKD 5](#_Toc148017074)

[Table S2D: Reclassification of male participants from CKD-EPI 2009 to EKFC CKD – NON-CKD 6](#_Toc148017075)

[2. Survival curves for the primary outcome 7](#_Toc148017076)

[3. Added value of eGFR over and above baseline risk prediction for the primary outcome 9](#_Toc148017077)

[Addition of CKD-EPI-2009 10](#_Toc148017078)

[Addition of CKD-EPI-2021 12](#_Toc148017079)

[Addition of EKFC 14](#_Toc148017080)

[Conclusion 15](#_Toc148017081)

[4. Association of CKD reclassification with the primary composite outcome 16](#_Toc148017082)

[(a). Association of reclassification to CKD-EPI 2021. 16](#_Toc148017083)

[(b). Association of reclassification to EKFC. 16](#_Toc148017084)

# Confusion matrices

## Table S1A: Reclassification of female participants from CKD-EPI 2009 to CKD-EPI 2021 KDIGO GFR categories

|  | **Events** | | | | | | **No events** | | | | | |
| --- | --- | --- | --- | --- | --- | --- | --- | --- | --- | --- | --- | --- |
|  | **NRIevents:** -3.5 %  (95 % CI: -9.1 % to 1.8 %) | | | | | | **NRInonevents**: -0.8 %  (95 % CI: -2.3 % to 0.7 %) | | | | | |
|  | **CKD-EPI 2021** | | | | | | **CKD-EPI 2021** | | | | | |
|  | **G1** | **G2** | **G3a** | **G3b** | **G4** | **G5** | **G1** | **G2** | **G3a** | **G3b** | **G4** | **G5** |
| **CKD-EPI 2009** | | | | | | | | | | | | |
| **G1** | 18 | 2 | 0 | 0 | 0 | 0 | 503 | 156 | 0 | 0 | 0 | 0 |
| **G2** | 6 | 64 | 4 | 0 | 0 | 0 | 133 | 1350 | 91 | 0 | 0 | 0 |
| **G3a** | 0 | 5 | 35 | 1 | 0 | 0 | 0 | 109 | 398 | 52 | 0 | 0 |
| **G3b** | 0 | 0 | 2 | 16 | 1 | 0 | 0 | 0 | 38 | 226 | 12 | 0 |
| **G4** | 0 | 0 | 0 | 1 | 11 | 0 | 0 | 0 | 0 | 8 | 58 | 1 |
| **G5** | 0 | 0 | 0 | 0 | 0 | 0 | 0 | 0 | 0 | 0 | 1 | 0 |

Participants are classified according to their KDIGO GFR category according to the GFR estimation following the CKD-EPI 2009 and the CKD-EPI 2021 equation, separated for “events” and “no events”. All those written in green are reclassified to a more suitable category according to their “event – no event status”, all those written in red are reclassified falsely according to their “event – no event status”. G1: eGFR ≥ 90 ml/min/1.73 m²; G2: eGFR = 60 – 89 ml/min/1.73 m²; G3a: eGFR = 45 – 59 ml/min/1.73 m²; G3b = 30 – 44 ml/min/1.73 m²; G4 = eGFR 15 – 29 ml/min/1.73 m²; G5 = eGFR < 15 ml/min/1.73 m². eGFR: estimated glomerular filtration rate, NRI: net reclassification improvement, CI: confidence interval.

## Table S1B: Reclassification of female participants from CKD-EPI 2009 to EKFC KDIGO GFR categories

|  | **Events** | | | | | | **No events** | | | | | |
| --- | --- | --- | --- | --- | --- | --- | --- | --- | --- | --- | --- | --- |
|  | **NRIevents:** 36.5 %  (95 % CI: 29.4 % to 44.3 %) | | | | | | **NRInonevents**: -37.4 %  (95 % CI: -39.0 % to -35.7 %) | | | | | |
|  | **EKFC** | | | | | | **EKFC** | | | | | |
|  | **G1** | **G2** | **G3a** | **G3b** | **G4** | **G5** | **G1** | **G2** | **G3a** | **G3b** | **G4** | **G5** |
| **CKD-EPI 2009** | | | | | | | | | | | | |
| **G1** | 3 | 17 | 0 | 0 | 0 | 0 | 179 | 480 | 0 | 0 | 0 | 0 |
| **G2** | 0 | 49 | 25 | 0 | 0 | 0 | 0 | 1140 | 434 | 0 | 0 | 0 |
| **G3a** | 0 | 0 | 27 | 14 | 0 | 0 | 0 | 0 | 348 | 211 | 0 | 0 |
| **G3b** | 0 | 0 | 0 | 14 | 5 | 0 | 0 | 0 | 0 | 231 | 45 | 0 |
| **G4** | 0 | 0 | 0 | 0 | 12 | 0 | 0 | 0 | 0 | 0 | 65 | 2 |
| **G5** | 0 | 0 | 0 | 0 | 0 | 0 | 0 | 0 | 0 | 0 | 0 | 1 |

Participants are classified according to their KDIGO GFR category according to the GFR estimation following the CKD-EPI 2009 and the EKFC equation, separated for “events” and “no events”. All those written in green are reclassified to a more suitable category according to their “event – no event status”, all those written in red are reclassified falsely according to their “event – no event status”. G1: eGFR ≥90 ml/min/1.73 m²; G2: eGFR 60-89 ml/min/1.73 m²; G3a: eGFR 45-59 ml/min/1.73 m²; G3b: 30-44 ml/min/1.73 m²; G4: eGFR 15-29 ml/min/1.73 m²; G5: eGFR <15 ml/min/1.73 m². eGFR: estimated glomerular filtration rate, NRI: net reclassification improvement, CI: confidence interval.

## Table S1C: Reclassification of female participants from CKD-EPI 2009 to CKD-EPI 2021 CKD – NON-CKD

|  | **Events** | | **No events** | |
| --- | --- | --- | --- | --- |
|  | **NRIevents: -**1.2 %  (95 % CI: -3.8 % to 1.2 %) | | **NRInonevents**: -0.4 %  (95 % CI: -1.2 % to 0.4 %) | |
|  | **CKD-EPI 2021** | | **CKD-EPI 2021** | |
|  | **CKD** | **NON-CKD** | **CKD** | **NON-CKD** |
| **CKD-EPI 2009** | | | | |
| **CKD** | 97 | 1 | 1055 | 86 |
| **NON-CKD** | 3 | 62 | 74 | 1757 |

Participants are classified according to the definition of CKD and NON-CKD according to the GFR estimation following the CKD-EPI 2009 and the CKD-EPI 2021 equation, separated for “events” and “no events”. All those written in green are reclassified to a more suitable group according to their “event – no event status”, all those written in red are reclassified falsely according to their “event – no event status”. CKD: chronic kidney disease, defined as eGFR <60 ml/min/1.73 m² or eGFR >60 ml/min/1.73 m² and albumin-to-creatinine ratio ≥30 mg/g, NRI: net reclassification improvement, CI: confidence interval.

## Table S1D: Reclassification of female participants from CKD-EPI 2009 to EKFC CKD – NON-CKD

|  | **Events** | | **No events** | |
| --- | --- | --- | --- | --- |
|  | **NRIevents:** -10.2 %  (95 % CI: -15.1 % to -5.9 %) | | **NRInonevents**: 11.4 %  (95 % CI: 10.3 % to 12.6 %) | |
|  | **EKFC** | | **EKFC** | |
|  | **CKD** | **NON-CKD** | **CKD** | **NON-CKD** |
| **CKD-EPI 2009** | | | | |
| **CKD** | 98 | 0 | 1141 | 0 |
| **NON-CKD** | 17 | 48 | 339 | 1492 |

Participants are classified according to the definition of CKD and NON-CKD according to the GFR estimation following the CKD-EPI 2009 and the EKFC equation, separated for “events” and “no events”. All those written in green are reclassified to a more suitable group according to their “event – no event status”, all those written in red are reclassified falsely according to their “event – no event status”. CKD: chronic kidney disease, defined as eGFR <60 ml/min/1.73 m² or eGFR >60 ml/min/1.73 m² and albumin-to-creatinine ratio ≥30 mg/g. EKFC: European Kidney Function Consortium, NRI: net reclassification improvement, CI: confidence interval.

## Table S2A: Reclassification of male participants from CKD-EPI 2009 to CKD-EPI 2021 KDIGO GFR categories

|  | **Events** | | | | | | **No events** | | | | | |
| --- | --- | --- | --- | --- | --- | --- | --- | --- | --- | --- | --- | --- |
|  | **NRIevents:** -12.0 %  (95 % CI: -16.4 % to -7.5 %) | | | | | | **NRInonevents**: 8.0 %  (95 % CI: 6.9 % to 9.1 %) | | | | | |
|  | **CKD-EPI 2021** | | | | | | **CKD-EPI 2021** | | | | | |
|  | **G1** | **G2** | **G3a** | **G3b** | **G4** | **G5** | **G1** | **G2** | **G3a** | **G3b** | **G4** | **G5** |
| **CKD-EPI 2009** | | | | | | | | | | | | |
| **G1** | 50 | 9 | 0 | 0 | 0 | 0 | 963 | 169 | 0 | 0 | 0 | 0 |
| **G2** | 25 | 146 | 3 | 0 | 0 | 0 | 384 | 2600 | 108 | 0 | 0 | 0 |
| **G3a** | 0 | 18 | 55 | 3 | 0 | 0 | 0 | 266 | 634 | 35 | 0 | 0 |
| **G3b** | 0 | 0 | 16 | 52 | 3 | 0 | 0 | 0 | 98 | 273 | 6 | 0 |
| **G4** | 0 | 0 | 0 | 6 | 7 | 0 | 0 | 0 | 0 | 17 | 59 | 0 |
| **G5** | 0 | 0 | 0 | 0 | 0 | 0 | 0 | 0 | 0 | 0 | 0 | 1 |

Participants are classified according to their KDIGO GFR category according to the GFR estimation following the CKD-EPI 2009 and the CKD-EPI 2021 equation, separated for “events” and “no events”. All those written in green are reclassified to a more suitable category according to their “event – no event status”, all those written in red are reclassified falsely according to their “event – no event status”. G1: eGFR ≥ 90 ml/min/1.73 m²; G2: eGFR = 60 – 89 ml/min/1.73 m²; G3a: eGFR = 45 – 59 ml/min/1.73 m²; G3b = 30 – 44 ml/min/1.73 m²; G4 = eGFR 15 – 29 ml/min/1.73 m²; G5 = eGFR < 15 ml/min/1.73 m². eGFR: estimated glomerular filtration rate, NRI: net reclassification improvement, CI: confidence interval.

## Table S2B: Reclassification of male participants from CKD-EPI 2009 to EKFC KDIGO GFR categories

|  | **Events** | | | | | | **No events** | | | | | |
| --- | --- | --- | --- | --- | --- | --- | --- | --- | --- | --- | --- | --- |
|  | **NRIevents:** 29.0 %  (95 % CI: 24.7 % to 33.5 %) | | | | | | **NRInonevents**: -27.5 %  (95 % CI: -28.7 % to -26.4 %) | | | | | |
|  | **EKFC** | | | | | | **EKFC** | | | | | |
|  | **G1** | **G2** | **G3a** | **G3b** | **G4** | **G5** | **G1** | **G2** | **G3a** | **G3b** | **G4** | **G5** |
| **CKD-EPI 2009** | | | | | | | | | | | | |
| **G1** | 15 | 44 | 0 | 0 | 0 | 0 | 399 | 733 | 0 | 0 | 0 | 0 |
| **G2** | 0 | 140 | 34 | 0 | 0 | 0 | 0 | 2516 | 576 | 0 | 0 | 0 |
| **G3a** | 0 | 0 | 51 | 25 | 0 | 0 | 0 | 0 | 723 | 212 | 0 | 0 |
| **G3b** | 0 | 0 | 0 | 60 | 11 | 0 | 0 | 0 | 0 | 350 | 27 | 0 |
| **G4** | 0 | 0 | 0 | 0 | 13 | 0 | 0 | 0 | 0 | 1 | 75 | 0 |
| **G5** | 0 | 0 | 0 | 0 | 0 | 0 | 0 | 0 | 0 | 0 | 0 | 1 |

Participants are classified according to their KDIGO GFR category according to the GFR estimation following the CKD-EPI 2009 and the EKFC equation, separated for “events” and “no events”. All those written in green are reclassified to a more suitable category according to their “event – no event status”, all those written in red are reclassified falsely according to their “event – no event status”. G1: eGFR ≥90 ml/min/1.73 m²; G2: eGFR 60-89 ml/min/1.73 m²; G3a: eGFR 45-59 ml/min/1.73 m²; G3b: 30-44 ml/min/1.73 m²; G4: eGFR 15-29 ml/min/1.73 m²; G5: eGFR <15 ml/min/1.73 m². eGFR: estimated glomerular filtration rate, NRI: net reclassification improvement, CI: confidence interval.

## Table S2C: Reclassification of male participants from CKD-EPI 2009 to CKD-EPI 2021 CKD – NON-CKD

|  | **Events** | | **No events** | |
| --- | --- | --- | --- | --- |
|  | **NRIevents:** 3.1 %  (95 % CI: 1.5 % to 4.9 %) | | **NRInonevents**: -2.3 %  (95 % CI: -3.0 % to -1.8 %) | |
|  | **CKD-EPI 2021** | | **CKD-EPI 2021** | |
|  | **CKD** | **NON-CKD** | **CKD** | **NON-CKD** |
| **CKD-EPI 2009** | | | | |
| **CKD** | 198 | 12 | 1704 | 209 |
| **NON-CKD** | 0 | 174 | 84 | 3370 |

Participants are classified according to the definition of CKD and NON-CKD according to the GFR estimation following the CKD-EPI 2009 and the CKD-EPI 2021 equation, separated for “events” and “no events”. All those written in green are reclassified to a more suitable group according to their “event – no event status”, all those written in red are reclassified falsely according to their “event – no event status”. CKD: chronic kidney disease, defined as eGFR <60 ml/min/1.73 m² or eGFR >60 ml/min/1.73 m² and albumin-to-creatinine ratio ≥30 mg/g, NRI: net reclassification improvement, CI: confidence interval.

## Table S2D: Reclassification of male participants from CKD-EPI 2009 to EKFC CKD – NON-CKD

|  | **Events** | | **No events** | |
| --- | --- | --- | --- | --- |
|  | **NRIevents:** -6.5 %  (95 % CI: -9.0 % to -4.1 %) | | **NRInonevents**: 8.3 %  (95 % CI: 7.6 % to 9.0 %) | |
|  | **EKFC** | | **EKFC** | |
|  | **CKD** | **NON-CKD** | **CKD** | **NON-CKD** |
| **CKD-EPI 2009** | | | | |
| **CKD** | 210 | 0 | 1913 | 0 |
| **NON-CKD** | 25 | 149 | 446 | 3008 |

Participants are classified according to the definition of CKD and NON-CKD according to the GFR estimation following the CKD-EPI 2009 and the EKFC equation, separated for “events” and “no events”. All those written in green are reclassified to a more suitable group according to their “event – no event status”, all those written in red are reclassified falsely according to their “event – no event status”. CKD: chronic kidney disease, defined as eGFR <60 ml/min/1.73 m² or eGFR >60 ml/min/1.73 m² and albumin-to-creatinine ratio ≥30 mg/g. EKFC: European Kidney Function Consortium, NRI: net reclassification improvement, CI: confidence interval.

.

# 2. Survival curves for the primary outcome


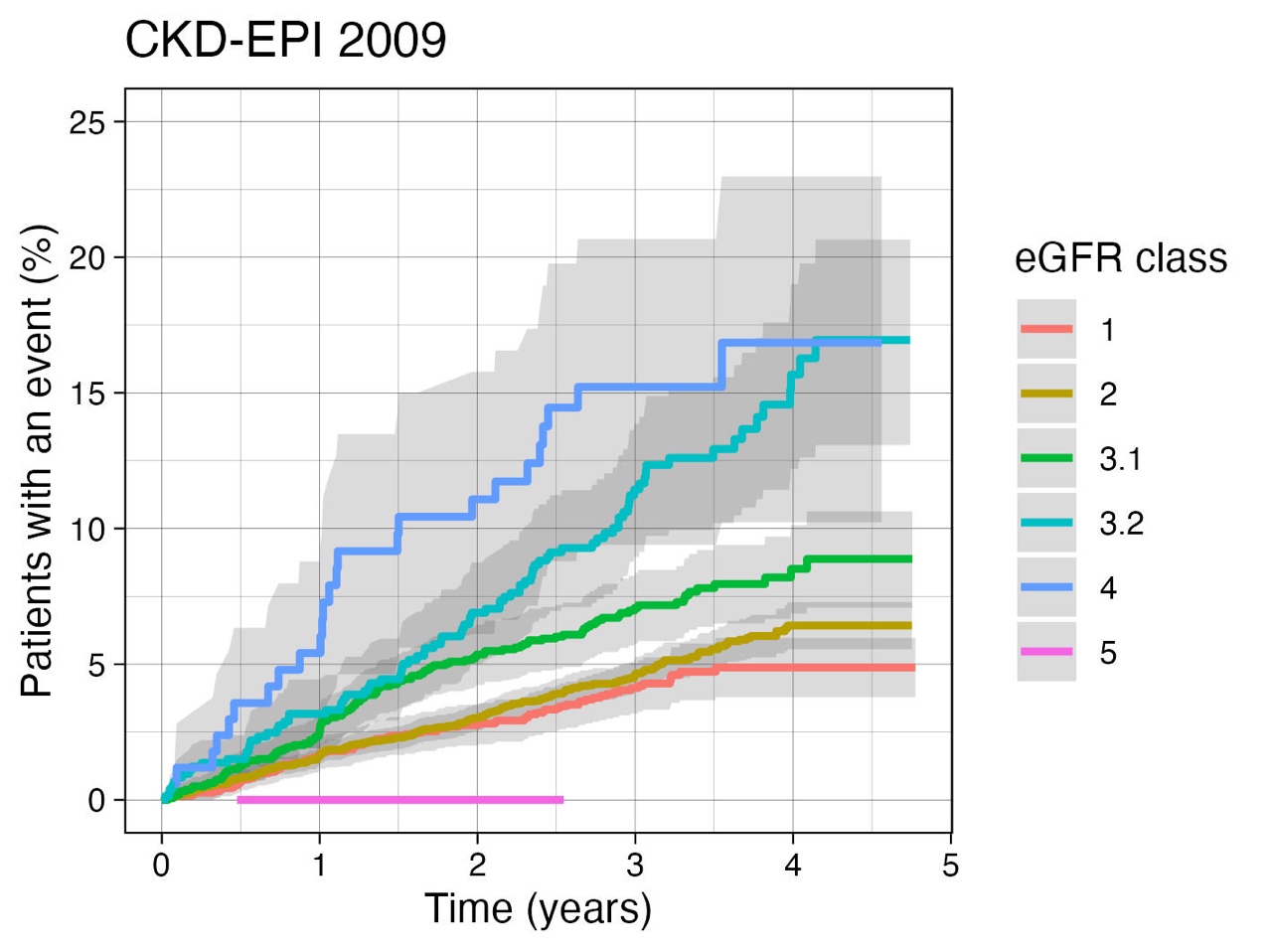


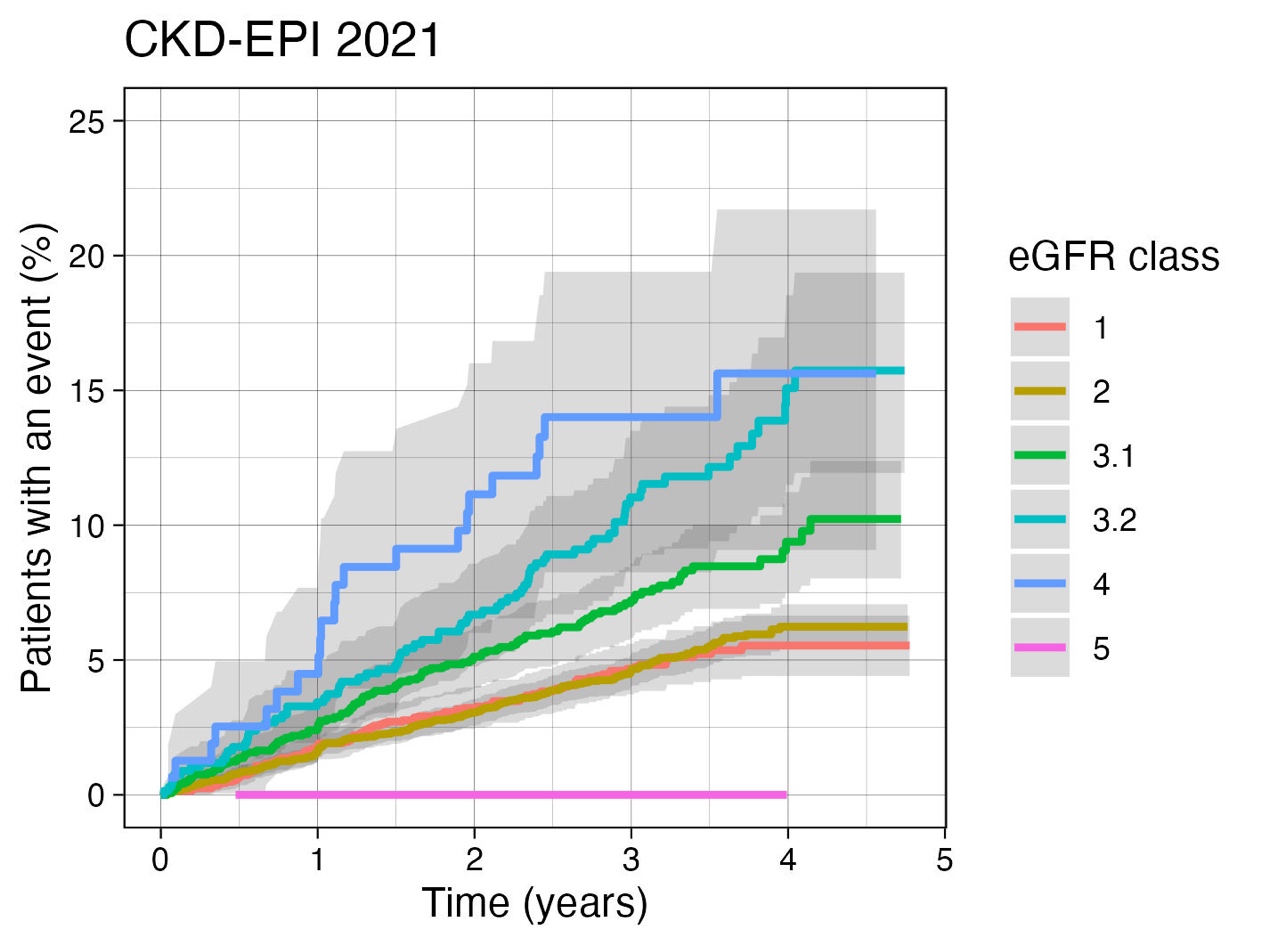


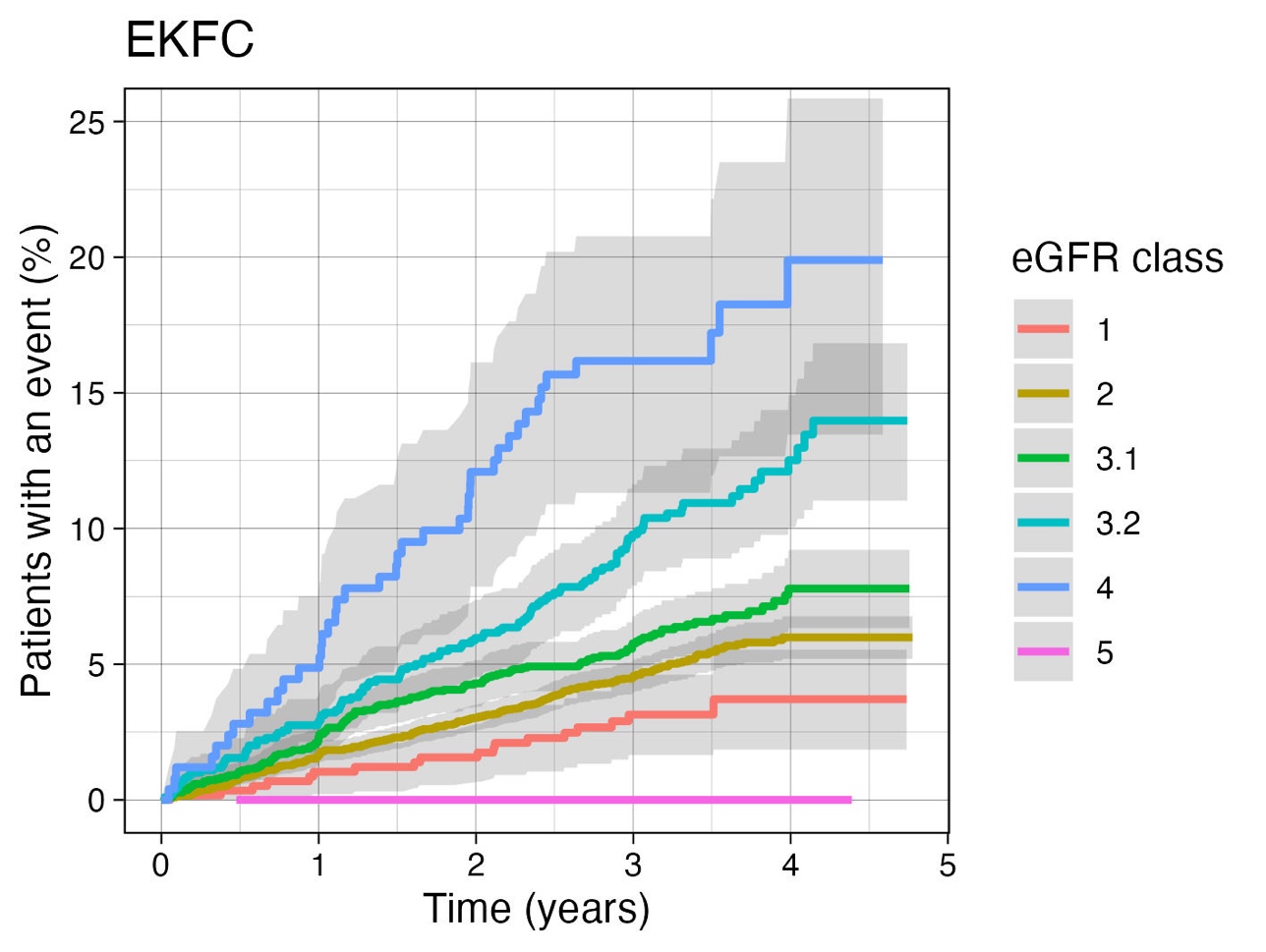


# 3. Added value of eGFR over and above baseline risk prediction for the primary outcome

This additional analysis uses statistical metrics and graphical techniques to ask if eGFR, by any of the equations, improves to the risk prediction for the primary outcome over and above a baseline model. A logistic regression model that truncates data collection at two years following recruitment to the SPRINT study is used.

The baseline model was:

Primary outcome ~ systolic blood pressure + diastolic blood pressure + smoking status + aspirin use + body mass index + high density cholesterol + log transformed triglyceride + total cholesterol + serum glucose + albumin to creatinine ratio + study arm

The performance of the baseline model was compared to **new** models that included each of the eGFR equations. The metrics presented are the Brier skill (relative change in Brier score), difference in area under the receiver operator characteristic curve (δAUC), Integrated Discrimination Improvement (IDI) for those who had the event (IDI_event_) and those who did not have the event separately (IDI_non-event_). Graphical assessment is given by risk assessment plots (RAP), decision curves, and calibration plots. Brier scores are a measure of the explained by the model variation in risk prediction from the actual outcomes and the Brier skill measures the improvement in this explanation with the addition of a biomarker. The AUC is the probability that if we were to draw at random a person who had an event and a person who did not have an event that the risk prediction for the person who had an event would be greater than for the person who did not have the event. The IDI_event_ represents the mean increase in risk prediction for those who had the event whereas the IDI_non-event_ represents the mean decrease in risk prediction for those who did not have the event. RAP plot Sensitivity verse risk prediction and 1-Specificity verse risk prediction. Improved performance with the addition of a biomarker would be observed by increased separation of the curves. Decision curves enable the assessment of the additional net benefit of BNP at specific risk thresholds of relevance to the clinician and patient.

Calculations and plots were made in R using the *rap* package available at: <https://github.com/JohnPickering/rap>.

## Addition of CKD-EPI-2009

| **Metric** | **Statistic (95%CI)** |
| --- | --- |
| Brier Skill | 0.29 (0.06 to 0.53) |
| AUC (baseline) | 0.680 (0.652 to 0.706) |
| AUC (new) | 0.681 (0.651 to 0.708) |
| δAUC | 0.001 (-0.008 to 0.009) |
| IDI_event_ | 0.002 (0.001 to 0.003) |
| IDI_non-event_ | 0 (0 to 0) |


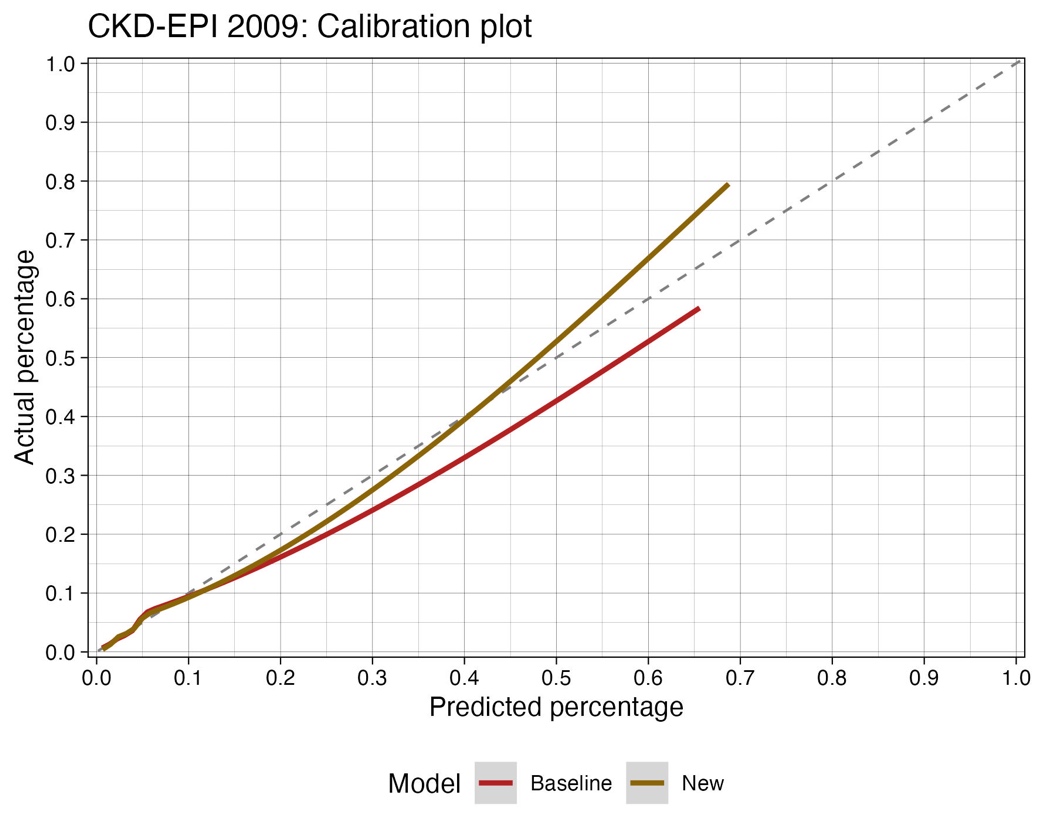


Figure 1: Calibration plot of the actual percentage of events verse that predicted by the models.


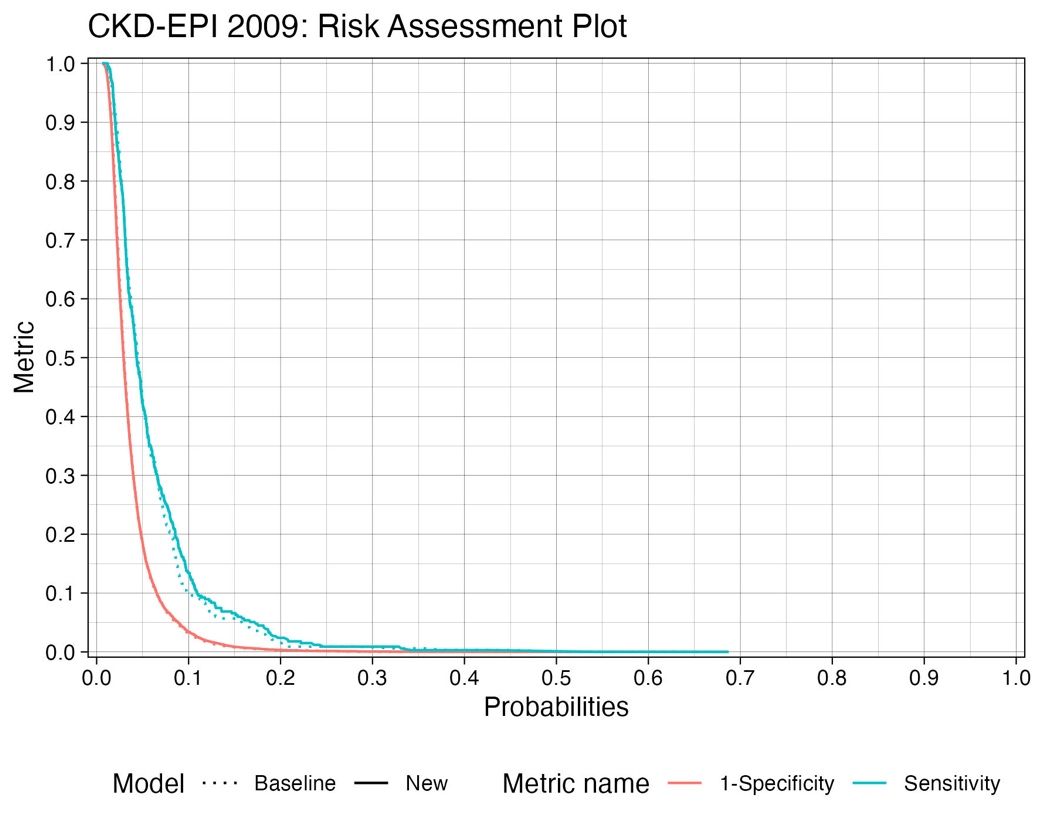


Figure 2: Risk assessment plot. Dotted lines are the baseline model senstivity (teal) verse probability of event and 1-specificity (red) verse probability of event. Solid lines are the new model. Improved performance would show the solid teal line moving towards the top right indicating increased probability of event in those who had the event, and the solid red line moving towards the bottom left indicating degreased probability of an event in those who did not have an event.


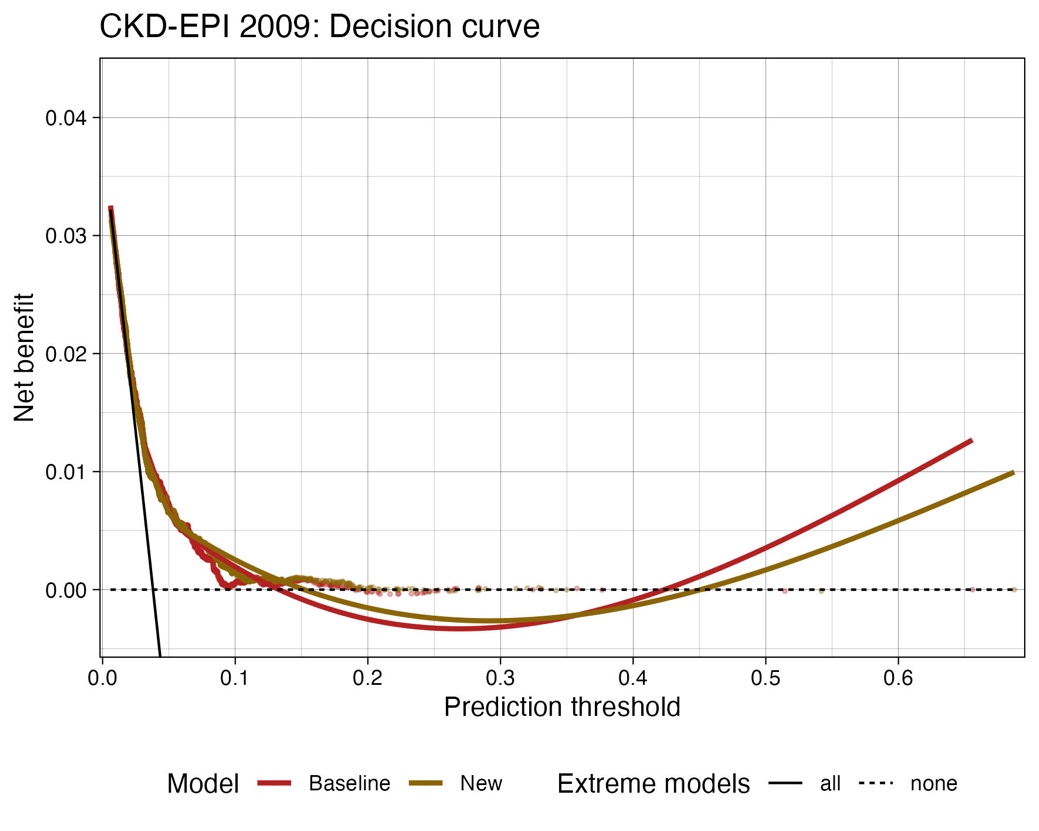


Figure 3: Decision curves, at any prediction threshold the further the new model is above the baseline model above the baseline, the better the model. Note, there were so few predictions > 0.4 that the curves are not indicative of model differences above that value.

## Addition of CKD-EPI-2021

| **Metric** | **Statistic (95%CI)** |
| --- | --- |
| Brier Skill | 0.24 (0.04 to 0.45) |
| AUC (baseline) | 0.679 (0.650 to 0.709) |
| AUC (new) | 0.678 (0.650 to 0.707) |
| δAUC | -0.001 (-0.008 to 0.006) |
| IDI_event_ | 0.002 (0.001 to 0.003) |
| IDI_non-event_ | 0 (0 to 0) |


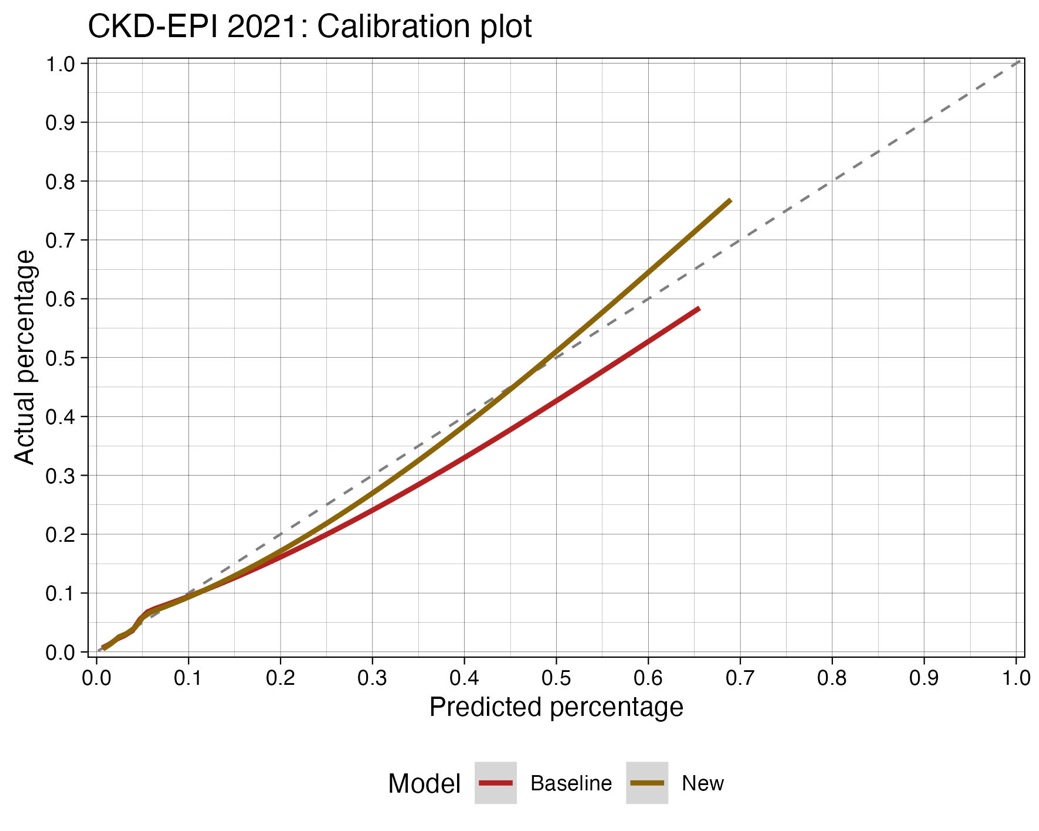


Figure 1: Calibration plot of the actual percentage of events verse that predicted by the models.


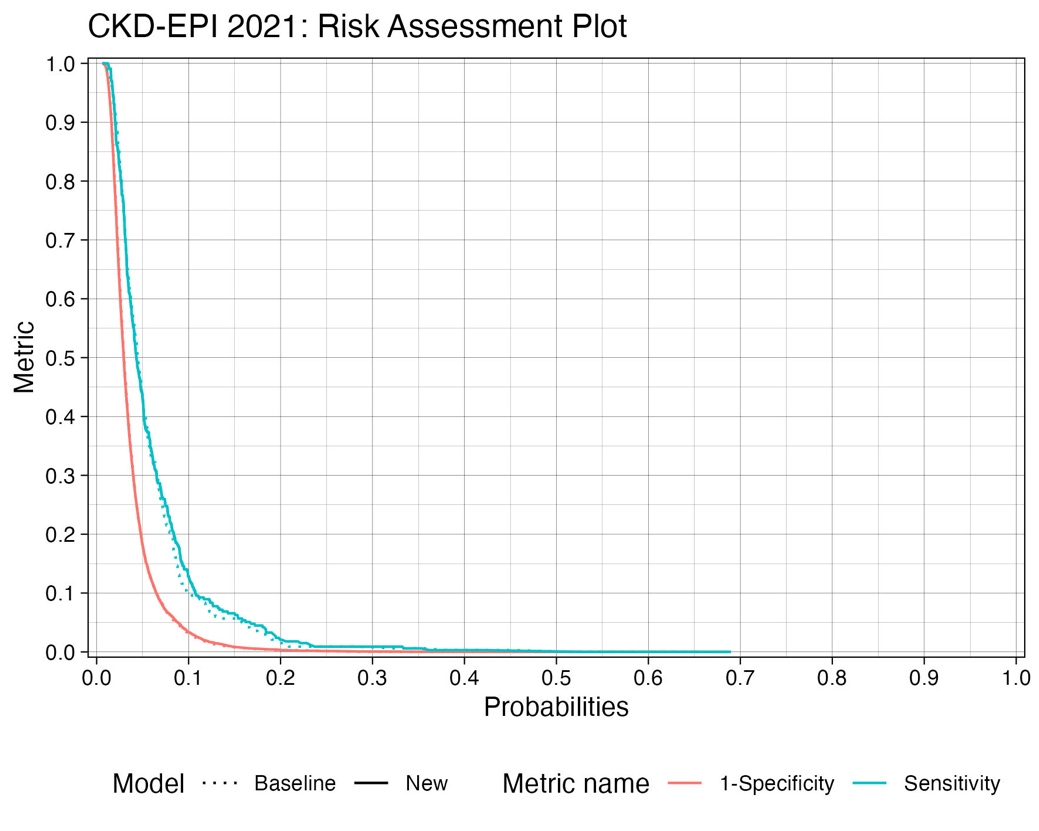


Figure 2: Risk assessment plot. Dotted lines are the baseline model sensitivity (teal) verse probability of event and 1-specificity (red) verse probability of event. Solid lines are the new model. Improved performance would show the solid teal line moving towards the top right indicating increased probability of event in those who had the event, and the solid red line moving towards the bottom left indicating degreased probability of an event in those who did not have an event.


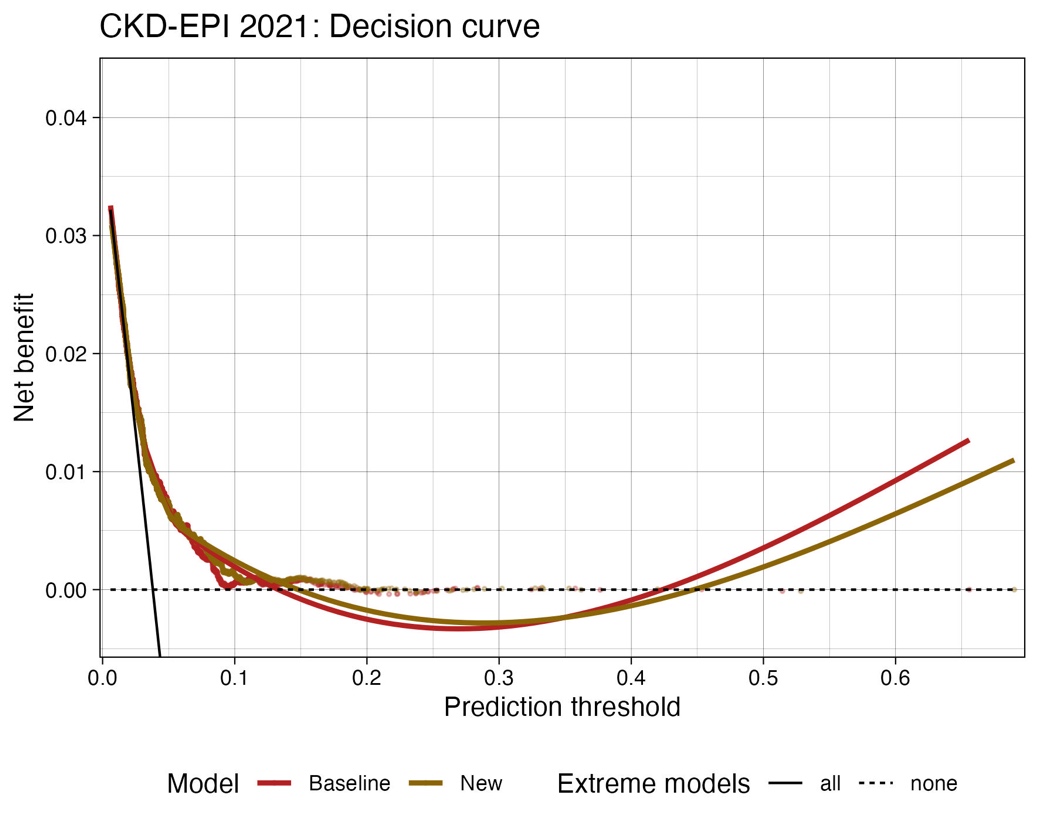


Figure 3: Decision curves, at any prediction threshold the further the new model is above the baseline model above the baseline, the better the model. Note, there were so few predictions > 0.4 that the curves are not indicative of model differences above that value.

## Addition of EKFC

| **Metric** | **Statistic (95%CI)** |
| --- | --- |
| Brier Skill | 0.30 (0.06 to 0.55) |
| AUC (baseline) | 0.679 (0.651 to 0.708) |
| AUC (new) | 0.680 (0.653 to 0.708) |
| δAUC | 0.001 (-0.009 to 0.01) |
| IDI_event_ | 0.002 (0.001 to 0.004) |
| IDI_non-event_ | 0 (0 to 0) |


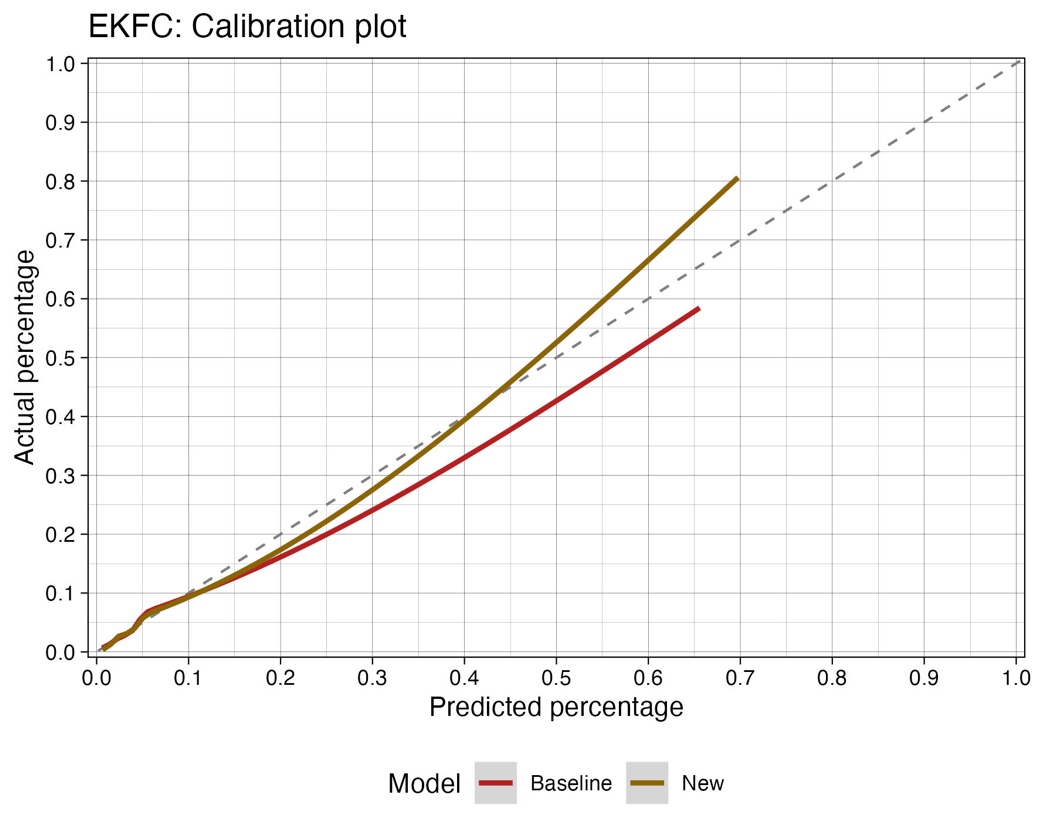


Figure 1: Calibration plot of the actual percentage of events verse that predicted by the models.


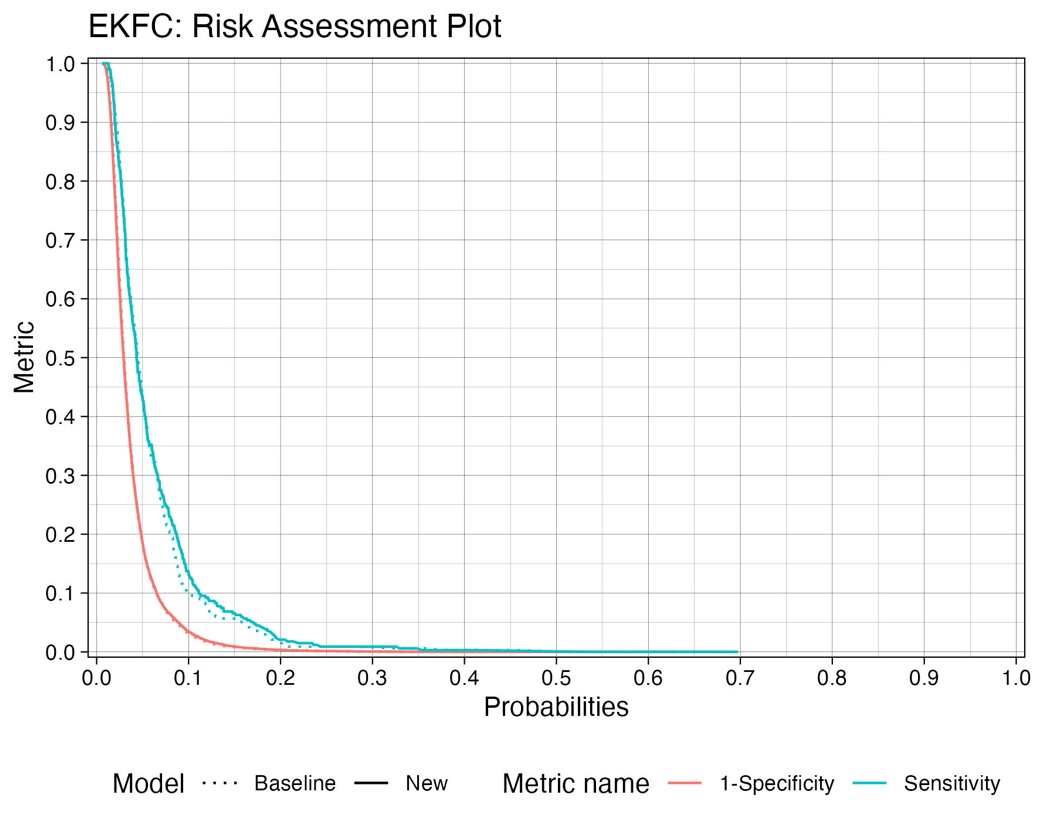


Figure 2: Risk assessment plot. Dotted lines are the baseline model sensitivity (teal) verse probability of event and 1-specificity (red) verse probability of event. Solid lines are the new model. Improved performance would show the solid teal line moving towards the top right indicating increased probability of event in those who had the event, and the solid red line moving towards the bottom left indicating degreased probability of an event in those who did not have an event.


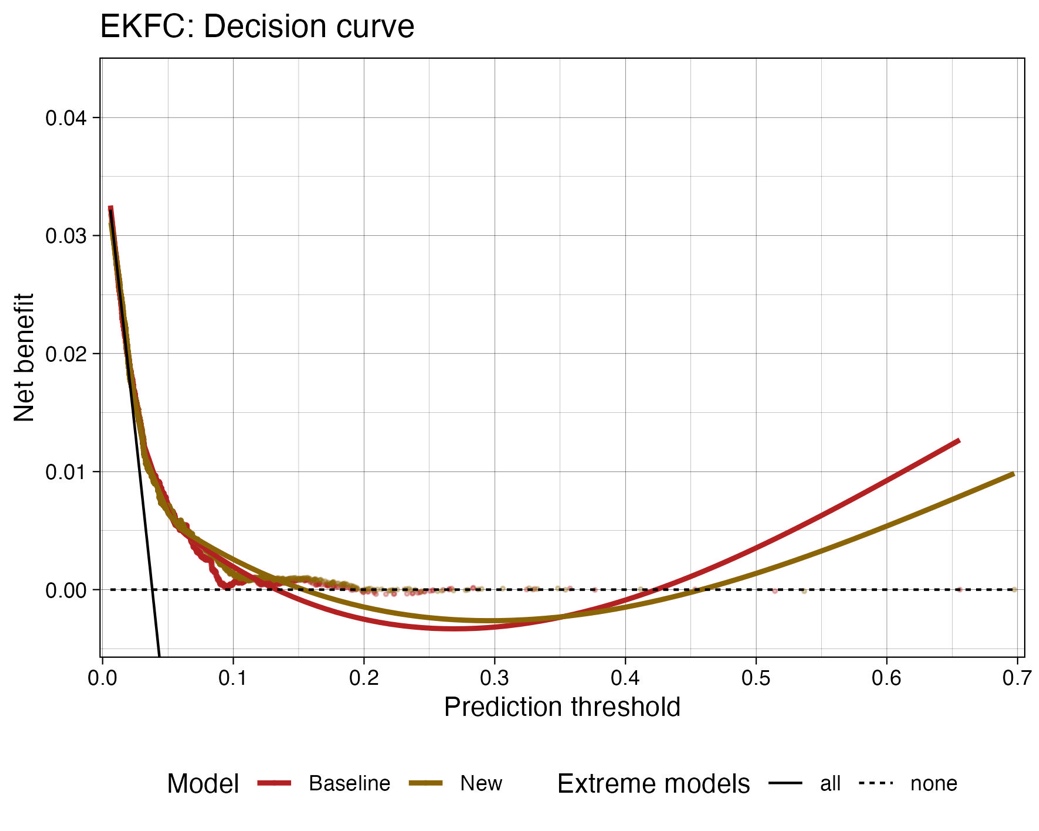


Figure 3: Decision curves, at any prediction threshold the further the new model is above the baseline model above the baseline, the better the model. Note, there were so few predictions > 0.4 that the curves are not indicative of model differences above that value.

# 4. Association of CKD reclassification with the primary composite outcome

Logistic regression model of:

primary outcome ~ Change in CKD status

## (a). Association of reclassification to CKD-EPI 2021.

The change in CKD status was a categorical variable with four classes:

0. Not CKD with both CKD-EPI 2009 and CKD-EPI 2021 (the reference)

1. CKD with CKD-EPI 2009 changed to Not CKD with CKD-EPI 2021

2. Not CKD with CKD-EPI 2009 changed to CKD with CKD-EPI 2021

3. CKD with both CKD-EPI 2009 and CKD-EPI 2021

| **Coefficient** | **Levels** | **Odds Ratio (95%CI)** | **P** |
| --- | --- | --- | --- |
| Change in CKD status | 0. Both not CKD | 1 (reference) |  |
|  | 1. CKD to Not CKD | 1.0 (0.5 to 1.7) | 0.89 |
|  | 2. Not CKD to CKD | 0.4 (0.1 to 1.3) | 0.13 |
|  | 3. Both CKD | 2.3 (1.9 to 2.8) | <0.001 |

## (b). Association of reclassification to EKFC.

The change in CKD status was a categorical variable with four classes:

0. Not CKD with both CKD-EPI 2009 and EKFC (the reference)

1. CKD with CKD-EPI 2009 changed to Not CKD with EKFC

2. Not CKD with CKD-EPI 2009 changed to CKD with EKFC

3. CKD with both CKD-EPI 2009 and EKFC

| **Coefficient** | **Levels** | **Odds Ratio (95%CI)** | **P** |
| --- | --- | --- | --- |
| Change in CKD status | 0. Both not CKD | 1 (reference) |  |
|  | 1. CKD to Not CKD | NA (no one reclassified) |  |
|  | 2. Not CKD to CKD | 1.2 (0.87 to 1.7) | 0.25 |
|  | 3. Both CKD | 2.3 (1.9 to 2.8) | <0.001 |

# 5. Characteristics of the eGFR equations in the SPRINT cohort

| Table S5: Equations’ characteristics | | | |
| --- | --- | --- | --- |
| **Name** | **Year of publication** | **Equations’ variables** | **Cohort summary of development data set** |
| CKD-EPI | 2009 | serum creatinine, age, race, sex, | N=8254; mean age: 47.0 ± 14.8 years; mean mGFR: 67.6 ± 39.6 ml/min/1.73 m²; mean SCr: 1.66 ± 1.16 mg/dl; 56.3 % males |
| CKD-EPI | 2021 | serum creatinine, age, sex | N=8254; mean age: 47.0 ± 14.8 years; mean mGFR: 67.6 ± 39.6 ml/min/1.73 m²; mean SCr: 1.66 ± 1.16 mg/dl; 56.3 % males |
| EKFC | 2020 | serum creatinine, age, sex | N=8473; mean age: 42.4 ± 25.2 years; mean mGFR: 76.9 ± 33.1 ml/min/1.73 m²; mean SCr/Q: 1.43 ± 0.91; 55.8 % males |

CKD – EPI = Chronic Kidney Disease Epidemiology Collaboration; EKFC = European Kidney Function Consortium; mGFR = measured glomerular filtration rate; SCr = serum creatinine; Q = scaling or normalization factor for SCr, representing the median SCr for the age / sex specific healthy population
